# Racial and Ethnic Disparities in Health-Related Outcomes in Crohn’s Disease: Results From the National Health and Wellness Survey

**DOI:** 10.1093/crocol/otae021

**Published:** 2024-04-12

**Authors:** Sabree C Burbage, Kathryn L Krupsky, M Janelle Cambron-Mellott, Nate Way, Aarti A Patel, Julia J Liu

**Affiliations:** Population Health Research, Janssen Scientific Affairs, LLC, Horsham, PA, USA; Real-World Evidence, Cerner Enviza, an Oracle Company, Kansas City, MO, USA; Real-World Evidence, Cerner Enviza, an Oracle Company, Kansas City, MO, USA; Real-World Evidence, Cerner Enviza, an Oracle Company, Kansas City, MO, USA; Population Health Research, Janssen Scientific Affairs, LLC, Horsham, PA, USA; Division of Gastroenterology, Morehouse School of Medicine, Atlanta, GA, USA

**Keywords:** Crohn’s disease, inflammatory bowel disease, National Health and Wellness Survey, condition severity, race/ethnicity

## Abstract

**Background:**

Crohn’s disease (CD) is a chronic inflammatory condition affecting the entire gastrointestinal tract that is associated with significant humanistic, clinical, and economic burdens. Few studies have assessed the association between CD severity and patient-reported outcomes (PROs), healthcare resource utilization (HCRU), and medical costs; even fewer have examined differences in disease outcomes among patients of various racial/ethnic groups.

**Methods:**

In this cross-sectional study, sociodemographic data, PROs, and economic outcomes for participants with self-reported CD were collected from the National Health and Wellness Survey (2018–2020). Multivariable analyses were used to assess the association of CD severity and race/ethnicity with health-related quality of life (HRQoL), work productivity and activity impairment (WPAI), HCRU, and medical costs.

**Results:**

Analyses included 1077 participants with CD (818 non-Hispanic White, 109 non-Hispanic Black, and 150 Hispanic). Participants with self-reported moderate/severe CD reported significantly worse HRQoL and WPAI, greater HCRU, and higher medical costs than those with self-reported mild CD. Non-Hispanic Black participants reported better HRQoL and fewer healthcare provider visits than non-Hispanic White participants. There were no significant differences in PROs between non-Hispanic White and Hispanic groups. Interactions between race/ethnicity and CD severity emerged for some, but not all groups: Specifically, non-Hispanic Black participants with moderate/severe CD reported greater absenteeism and more gastroenterologist visits than non-Hispanic Black participants with mild CD.

**Conclusions:**

Participants with moderate/severe CD reported worse PROs, greater HCRU, and higher medical costs than those with mild CD. Additionally, racial/ethnic differences were found across several HCRU and economic outcomes. Further research is needed to better understand factors contributing to burden among patients with varying CD severity across racial/ethnic groups.

## Graphical Abstract

**Graphical Abstract:**
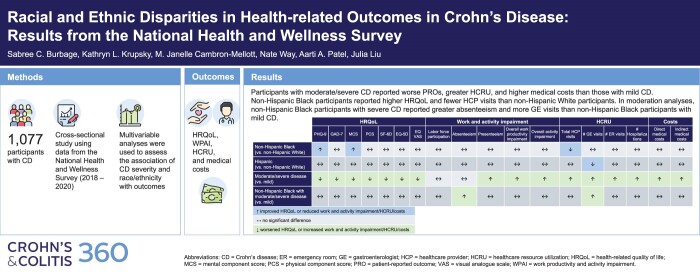

## Introduction

Crohn’s disease (CD), a type of inflammatory bowel disease (IBD), is a chronic inflammatory condition of the gastrointestinal (GI) tract that is characterized by relapsing and remitting symptoms of abdominal pain, diarrhea, bowel obstruction, weight loss, and fatigue.^1^ Among patients with multiple relapses, CD can quickly progress from a mild to moderate condition, and eventually cause severe penetrating or stricturing disease.^2^ Despite the development of new treatments for CD, including biologics and immunomodulators, up to 75% of patients with CD require surgery throughout their lifespan, with many requiring several surgical procedures.^3^

The CD has debilitating effects on patients’ health-related quality of life (HRQoL), which may be attributed to impairments in the ability to participate in social and economic/vocational opportunities and diminished psychological well-being.^1^ Most patients with CD develop symptoms before the age of 30, coinciding with their primary reproductive and working years.^1^ Feelings of social isolation and stress due to the unpredictable nature of symptoms are common and patients with CD have a higher-than-average incidence of depression and anxiety.^4^ Symptoms of CD can negatively influence work productivity, leading to higher rates of unemployment and work disability relative to the general population.^5^ CD is also associated with high rates of healthcare resource utilization (HCRU), including emergency room visits, hospitalizations, medical procedures, and surgeries.^6^ Although medication and hospitalization are the main drivers of cost for patients with CD, indirect costs due to lost productivity can also be high. A recent study estimated total annualized medical costs for CD patients ranging from $16 711 to $66 027.^6^ Importantly, moderate or severe CD symptoms are associated with significantly lower HRQoL, increased work productivity loss, greater HCRU, and higher medical costs than patients with mild symptoms.^7–10^

From 1970 to 2010, the incidence of IBD increased by 39% among White individuals and by 134% among nonwhite individuals globally.^11^ In the United States, the prevalence of IBD has historically been higher among White individuals than Black individuals.^12^ However, recent studies have reported similar incidence rates of IBD among White and Black individuals.^13^ The increasing incidence of CD among marginalized groups in the United States has brought forth awareness of differences in the disease course and patient journey.^14^ As such, understanding racial/ethnic differences is crucial for improving health equity and ensuring timely and adequate treatment of disease.

Patient-reported outcomes (PROs) are increasingly used in clinical studies as they emphasize the patient experience of disease burden.^15^ As IBD is an under-recognized condition in minority patient groups, PROs can allow data collection from a diverse patient population. Particularly, considering self-reported severity is crucial for understanding disease burden, as symptoms identified as most problematic by patients may not be considered the cardinal symptoms assessed in clinical practice.^16^ Furthermore, the impact of IBD symptoms on patients’ lives is often underestimated.^17,18^ There are currently limited studies on PROs in the context of CD severity and race/ethnicity among patients with CD, and even fewer studies that compare more than 2 race/ethnicity groups simultaneously. Therefore, the objective of this study was to evaluate the relationship of race/ethnicity and CD severity with PROs of depression, anxiety, HRQoL, and work productivity and activity impairment (WPAI), and health economic outcomes such as HCRU, medical costs, and indirect costs among non-Hispanic White, non-Hispanic Black, and Hispanic patients with mild or moderate/severe CD.

## Methods

### Study Design

This retrospective, cross-sectional study leveraged data from the 2018, 2019, and 2020 US National Health and Wellness Survey (NHWS). The NHWS is a self-administered, internet-based survey administered to a nationally representative sample of adults (ages ≥ 18 years; ~*N* = 75 000 per year).^19^ Potential participants were recruited through opt-in e-mails, co-registration with panel partners, e-newsletter campaigns, banner placements, and affiliate networks. All panelists agreed to take part as a panel member and completed an in-depth demographic registration profile. A quota sampling technique ensured that the demographic composition (ie, age, gender, and race/ethnicity) of the NHWS sample was representative of the adult population in the United States. The NHWS was reviewed by Pearl Institutional Review Board (Indianapolis, IN) and granted exemption status. All participants provided their informed consent electronically.

### Study Population

**Figure 1.**
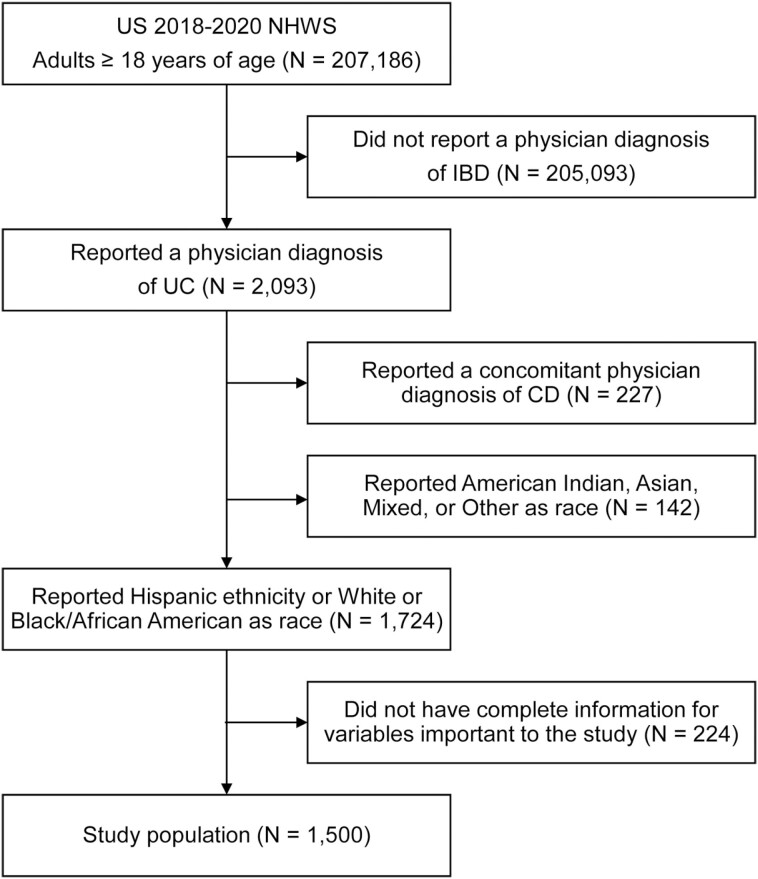
Study participant flow chart. Abbreviations: CD, Crohn’s disease; IBD, inflammatory bowel disease; NHWS, National Health and Wellness Survey; UC, ulcerative colitis; US, United States.

Participants were included in the analytic sample if they met the following inclusion criteria: ≥18 years old, resident of the United States, self-reported receipt of a physician diagnosis of CD, self-reported Hispanic ethnicity or non-Hispanic White or non-Hispanic Black/African American race and had complete data for outcomes relevant to the study (Figure 1). Participants who reported a concomitant diagnosis of ulcerative colitis or another race/ethnicity were excluded from the study sample.

### Sociodemographic and Clinical Characteristics

Self-reported sociodemographic and clinical characteristics collected from the NHWS were as follows: Sex, age, marital status, education level, employment status, household income, health insurance, CD severity, body mass index, alcohol use, cigarette smoking, exercise, current prescription medication use, sleep problems/symptoms, comorbidity index, patient activation measure (PAM) score,^20^ and previous clinical trial participation. Self-reported severity of condition was captured as participants’ rating of their CD as either mild, moderate, or severe. Participants who used a prescription medication for CD during the study period rated their CD severity while on their medication, whereas participants who did not use prescription medication rated their CD severity in a general sense (ie, not specific to any symptoms). Self-reported race and ethnicity were categorized into 3 mutually exclusive groups: Non-Hispanic White, non-Hispanic Black, or Hispanic. Patient activation (or engagement) was assessed using the PAM, a non-disease-specific measure that assesses an individual’s knowledge and confidence in managing their health.^20^ The questionnaire includes 13 items with a total score of 0 to 100, where higher scores indicate greater levels of engagement. Individuals can also be grouped into 4 levels of activation (ie, level 1: 0–47, level 2: 47.1–55.1, level 3: 55.2–72.4, and level 4: 72.5–100). Previous clinical trial participation data were collected from a non-disease-specific survey question; response options included “yes” or “no.”

### Study Outcomes

Outcomes of interest included PROs of depression and anxiety, HRQoL, labor force participation, WPAI, and economic outcomes of HCRU and medical costs. Detailed descriptions of the study outcomes and how they were calculated are included in Supplementary Materials. Depression severity was assessed using the patient health questionnaire-9 (PHQ-9) and anxiety severity was assessed using the generalized anxiety disorder assessment (GAD-7). HRQoL was assessed using 2 summary scores of the medical outcomes study 36-item short form survey instrument (SF-36v2): physical component summary (PCS) and mental component summary (MCS).^21^ The SF-6D and the EuroQol 5-dimension health questionnaire (EQ-5D) instruments were used to assess health state utilities, the latter of which consists of the EQ-5D-5L utility index and EQ visual analog scale (VAS).^22^ Labor force participation was derived from NHWS data through coding employment status as currently in the labor force (ie, full-time employed, part-time employed, self-employed, or not unemployed but looking for work) or not currently in the labor force (ie, retired, disabled, homemaker, student, or not employed and not looking for work). Work productivity was assessed using the WPAI questionnaire.^23^ Only participants who reported a work status of full-time, part-time, or self-employed provided data for absenteeism, presenteeism, and overall work impairment. HCRU included the number of self-reported visits to any healthcare provider (HCP), gastroenterologists (GE), and emergency rooms (ER), and hospitalizations over the past 6 months. Direct medical costs were imputed using data from the region-specific Medical Expenditure Panel Survey and included costs of an average HCP visit, ER visit, and hospitalization.^24^ Indirect costs were those associated with work productivity impairment and were calculated using estimated wages/salaries for each participant with data from the US Bureau of Labor Statistics. This cost analysis approach has been used in prior research in the NHWS.^25,26^

### Data Analysis

For analyses, due to sample size limitations with the severe CD category (Table 1), moderate and severe CD were grouped into 1 category to evaluate disease severity as an effect modifier. Descriptive statistics (frequencies, percentages for categorical variables; means with standard deviations [SD]) were used to characterize the CD cohort and better understand the distribution of key variables. Bivariate analyses were used to examine sociodemographic and clinical characteristics across CD severity groups (ie, mild and moderate/severe disease) and race/ethnicity groups (ie, non-Hispanic White, non-Hispanic Black, and Hispanic) among patients with self-reported physician-diagnosed CD and inform covariate selection for multivariable analyses (Supplementary Tables S1 and S3).

**Table 1.** Sociodemographic characteristics of participants with self-reported Crohn’s Disease.

	Non-Hispanic White	Non-Hispanic Black	Hispanic
	(*n* = 818)	(*n* = 109)	(*n* = 150)
Female, *n* (%)	421 (51.5%)	55 (50.5%)	55 (36.7%)^†^
Age in years, mean (SD)	48.34 (16.48)	36.84 (14.39)*	34.05 (11.05)^†^
Marital Status, *n* (%)			
Married/living with partner	518 (63.3%)	37 (33.9%)*	94 (62.7%)^‡^
Single, not married/divorced/separated/widowed	299 (36.6%)	71 (65.1%)*	56 (37.3%)^‡^
Decline to answer	1 (0.1%)	1 (0.9%)	0 (0.0%)
Education, *n* (%)			
Less than high school	6 (0.7%)	4 (3.7%)*	3 (2.0%)
Completed some high school	22 (2.7%)	5 (4.6%)	5 (3.3%)
High school graduate or equivalent (eg, GED)	90 (11.0%)	16 (14.7%)	30 (20.0%)^†^
Completed some college, but no degree	159 (19.4%)	26 (23.9%)	21 (14.0%)
Associate degree	106 (13.0%)	15 (13.8%)	22 (14.7%)
College graduate	222 (27.1%)	27 (24.8%)	36 (24.0%)
Completed some graduate school, but no degree	45 (5.5%)	2 (1.8%)	11 (7.3%)
Completed graduate school	167 (20.4%)	14 (12.8%)	22 (14.7%)
Decline to answer	1 (0.1%)	0 (0.0%)	0 (0.0%)
Employment status, *n* (%)			
Employed full-time	364 (44.5%)	56 (51.4%)	94 (62.7%)^†^
Self-employed	54 (6.6%)	9 (8.3%)	13 (8.7%)
Employed part-time	83 (10.1%)	14 (12.8%)	12 (8.0%)
Homemaker	39 (4.8%)	7 (6.4%)	3 (2.0%)
Retired	159 (19.4%)	2 (1.8%)*	9 (6.0%)^†^
Student	21 (2.6%)	9 (8.3%)*	7 (4.7%)
Long-term disability	56 (6.8%)	5 (4.6%)	5 (3.3%)
Not employed (whether looking for work or not)	42 (5.1%)	7 (6.4%)	7 (4.7%)
Household income, *n* (%)			
<$25 000	102 (12.5%)	32 (29.4%)*	16 (10.7%)^‡^
$25 000 to <$50 000	163 (19.9%)	20 (18.3%)	29 (19.3%)
$50 000 to <$100 000	289 (35.3%)	29 (26.6%)	47 (31.3%)
$100 000+	246 (30.1%)	27 (24.8%)	57 (38.0%)
Decline to answer	18 (2.2%)	1 (0.9%)	1 (0.7%)
Health insurance, *n* (%)			
Not insured	58 (7.1%)	16 (14.7%)*	21 (14.0%)^†^
Commercially insured	456 (55.7%)	65 (59.6%)	91 (60.7%)
Medicaid	81 (9.9%)	10 (9.2%)	11 (7.3%)
Medicare	203 (24.8%)	15 (13.8%)*	14 (9.3%)^†^
Other type of insurance/unsure	20 (2.4%)	3 (2.8%)	13 (8.7%)^†^
PAM Score^a^, mean (SD)	62.31 (12.30)	56.90 (13.95)*	58.16 (13.76)^†^
PAM Level^b^, *n* (%)			
Level 1	70 (8.6%)	17 (15.6%)	35 (23.3%)^†^
Level 2	134 (16.4%)	28 (25.7%)	29 (19.3%)
Level 3	475 (55.9%)	50 (45.9%)	64 (42.7%)^†^
Level 4	157 (19.2%)	14 (12.8%)	22 (14.7%)
Severity of condition^c^, *n* (%)			
Mild	514 (62.8%)	51 (46.8%)*	83 (55.3%)
Moderate	249 (30.4%)	43 (39.4%)	50 (33.3%)
Severe	55 (6.7%)	15 (13.8%)*	17 (11.3%)
Ever participated in any clinical trial^d^, *n* (%)	164 (20.0%)	37 (33.9%)*	68 (45.3%)^†^

^a^PAM scores range from 0 to 100 where higher scores indicate higher levels of activation.

^b^PAM scores correlate to 1 of the 4 levels of patient activation: Level 1 = disengaged and overwhelmed; Level 2 = becoming aware but still struggling; Level 3 = taking action; Level 4 = maintaining behavior and pushing further.

^c^Due to sample size limitations, moderate and severe CD were grouped into 1 category to allow for the evaluation of disease severity as an effect modifier.

^d^This refers to all clinical trials, not just IBD-related trials.

Sociodemographic characteristics were assessed using bivariate analyses.

^*^ *α *< 0.05 between non-Hispanic Black and non-Hispanic White participants. ^†^*α* < 0.05 between Hispanic and non-Hispanic White participants. ^‡^*α* < 0.05 between Hispanic and non-Hispanic Black participants. Results were adjusted for multiple comparisons with the Bonferroni correction.

Abbreviations: PAM, patient activation measure; SD, standard deviation.

To assess associations between CD severity and race/ethnicity with outcomes of interest, multivariable analyses were conducted by constructing generalized linear regression models using the outcome-appropriate distribution and link functions (ie, linear model for SF-36 and EQ-5D outcomes, binary logistic regression model for labor force participation, and negative binomial distribution with log-link for all other outcomes). The same covariates were included in each regression model for all outcomes, enabling comparisons of the magnitude of the association for each independent variable. Models controlled for age (continuous; set to mean = 45.19 years), sex (male [reference group (ref)] or female), marital status (single/never married/decline to answer or married/living with a partner [ref]), educational attainment (less than a college degree/declined to answer or college graduate or higher [ref]), household income (<$25 000, $25 000 to <$50 000, $50 000 to <$100 000, or $100 000 + [ref]), health insurance coverage (Medicare, Medicaid/VA/CHAMPUS, uninsured, or commercial/other types of insurance [ref]), weight status (obese, overweight, or underweight/normal weight/declined to answer [ref]), smoking status (current smoker, former smoker, or never smoker [ref]), alcohol use (drinks alcohol or does not drink alcohol [ref]), and Charlson Comorbidity Index Score (continuous; set to mean = 1.14). Adjusted means with standard error (SE), mean differences (MD), rate ratios (RR), and *P* values were reported.

Separate covariate-adjusted models were used to assess whether the relationship between CD severity and specific outcomes depended on race/ethnicity. Heterogeneity of the association was determined using an interaction term between race/ethnicity and CD severity, which was added to each covariate-adjusted model. The statistical significance of the interaction term was assessed using type III analyses (the F test for linear models and likelihood ratio test for logistic or negative binomial models). For models where the interaction term was statistically significant, interactions were probed via stratification, and covariate-adjusted estimates were interpreted in the context of the interaction term. All analyses were conducted using SAS 9.4.

## Results

### Sociodemographic and Clinical Characteristics

This study included 1077 participants with a self-reported diagnosis of CD from a physician. Of these participants, 60.2% reported having mild disease and 39.8% reported having moderate/severe disease; furthermore, 76.0% identified as non-Hispanic White, 13.9% as Hispanic, and 10.1% as non-Hispanic Black. There was a lower proportion of female participants in the Hispanic group (36.7%) than in the non-Hispanic White group (51.5%; *P = *.003) (Table 1). Mean age was lower among non-Hispanic Black (36.84 [SD: 14.39]) and Hispanic participants with CD (34.05 [SD: 11.05]) than among non-Hispanic White participants (48.34 [SD: 16.48]; both *P* < .001). Severe CD was reported by a higher proportion of non-Hispanic Black participants (13.8%) than Hispanic (11.3%) and non-Hispanic White participants (6.7%; *P* =* *.032), and mild disease was reported by a greater proportion of non-Hispanic White participants (62.8%) than Hispanic (55.3%) and non-Hispanic Black participants (46.8%; *P* = .004). Interestingly, only the non-Hispanic Black participant group had a greater proportion of moderate/severe disease than mild disease. A greater proportion of non-Hispanic Black participants had a household income <$25 000 (29.4%) than non-Hispanic White (12.5%; *P *< .001) and Hispanic (10.7%; *P* = .001) participants. Although a similar proportion of participants across race/ethnicity groups had commercial insurance, a lower proportion of non-Hispanic White participants were not insured (7.1%) than non-Hispanic Black (14.7%; *P *= .022) and Hispanic (14.0%; *P = *.016) participants.

Patient activation, on average, was lower among non-Hispanic Black (56.90 [SD: 13.95]) and Hispanic (58.16 [SD: 13.76]) participants than non-Hispanic White participants (62.31 [SD: 12.30]; *P* < .001 and *P* = .001, respectively; Table 1). Notably, 72.6% of the study population had a PAM level of 3 (ie, taking action and gaining control) or 4 (ie, maintaining behaviors and pushing further). Previous participation in any clinical trial was significantly higher among non-Hispanic Black (33.9%) and Hispanic participants (45.3%) than among non-Hispanic White participants (20.0%; *P* = .003 and *P *< .001, respectively).

### Study Outcomes by Disease Severity—Main Effects

**Figure 2.**
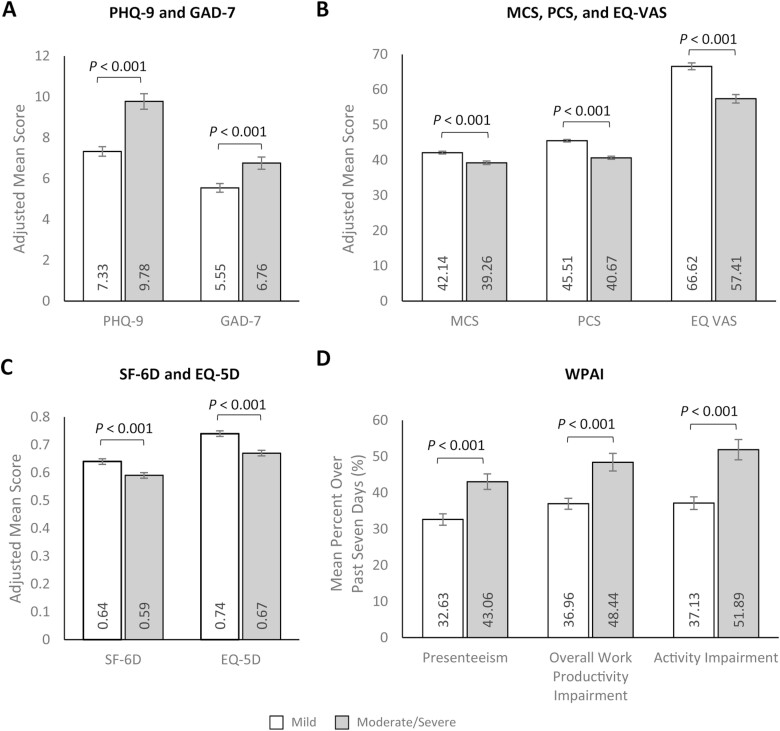
Multivariable analyses of patient-reported outcomes of (A) PHQ-9 and GAD-7, (B) MCS, PCS, and EQ VAS, (C) SF-6D and EQ 5D, and (D) WPAI by Crohn’s disease severity. Note: Lower PHQ-9 and GAD-7 scores and higher MCS, PCS, EQ VAS, SF-6D, and EQ-5D scores indicate better HRQoL. Error bars represent standard error. Presenteeism was only assessed among participants who worked > 0 hours over the last 7 days. PHQ-9, GAD-7, and WPAI outcomes were modeled using a log link with a negative binomial distribution; exp(β) = rate ratio. MCS, PCS, EQ VAS, SF-6D, and EQ-5D were modeled using an identity link with a normal distribution; β = estimated mean difference. Models control for age (continuous; set to mean = 45.19 years), gender (male [ref]; female), marital status (single/never married/decline to answer; married/living with a partner [ref]), educational attainment (less than a college degree/declined to answer; college graduate or higher [ref]), household income (<$25 000; $25 000 to <$50 000; $50 000 to <$100 000; $100 000 + [ref]), health insurance coverage (Medicare; Medicaid/VA/CHAMPUS; uninsured; commercial/other types of insurance [ref]), weight status (obese; overweight; underweight/normal weight/ declined to answer [ref]), smoking status (current smoker; former smoker; never smoker [ref]), alcohol use (drinks alcohol; does not drink alcohol [ref]), and Charlson Comorbidity Index Score (continuous; set to mean = 1.14). Abbreviations: EQ-5D-5L, EuroQol 5-Dimension 5-Level; GAD-7, generalized anxiety disorder—7 item; MCS, mental component summary; PCS, physical component summary; PHQ-9, patient health questionnaire-9 item; SF-6D, short form—6 dimension; WPAI, work productivity and activity impairment; VAS, visual analog scale.

Multivariable analyses adjusting for potential confounders showed that participants with moderate/severe CD had significantly worse self-reported depression and anxiety than those with mild CD (PHQ-9: 9.78 [SE: 0.38] vs. 7.33 [SE: 0.23]; *P* < .001 and GAD-7: 6.76 [SE: 0.30] vs. 5.55 [SE: 0.21]; *P* = .001; Figure 2A, Supplementary Table S1). Similarly, participants with moderate/severe CD had significantly worse HRQoL, as measured using MCS and PCS scores, EQ VAS, and utility index scores (ie, SF-6D and EQ-5D), than those with mild CD (MCS: 39.26 [SE: 0.49] vs. 42.14 [SE: 0.39]; MD: −2.87 [SE 0.63]; *P* < .001, PCS: 40.67 [SE: 0.43] vs. 45.51 [SE: 0.35]; MD: −4.84 [SE 0.56]; *P* < .001, EQ VAS: 57.41 [SE: 1.21] vs. 66.62 [0.98]; MD: −9.20 [1.57]; *P* < .001, SF-6D: 0.59 [SE: 0.01] vs. 0.64 [SE: 0.01]; MD: −0.06 [SE 0.01]; *P* < .001, and EQ-5D-5L: 0.67 [SE: 0.01] vs. 0.74 [SE: 0.01]; MD: −0.08 [SE: 0.01]; *P* < .001; Figure 2B and 2C, Supplementary Table S1).

Estimated WPAI scores showed that presenteeism, overall work productivity impairment, and overall activity impairment were higher among participants with moderate/severe CD than those with mild CD (presenteeism: 43.06% [SE: 2.43] vs. 32.63% [SE: 1.52]; *P* < .001; overall work productivity impairment: 48.44% [SE: 2.80] vs. 36.96% [SE: 1.76]; *P* < .001; overall activity impairment: 51.89% [SE: 1.86] vs. 37.13% [SE: 1.08]; *P *< .001; Figure 2D, Supplementary Table S1).

**Figure 3.**
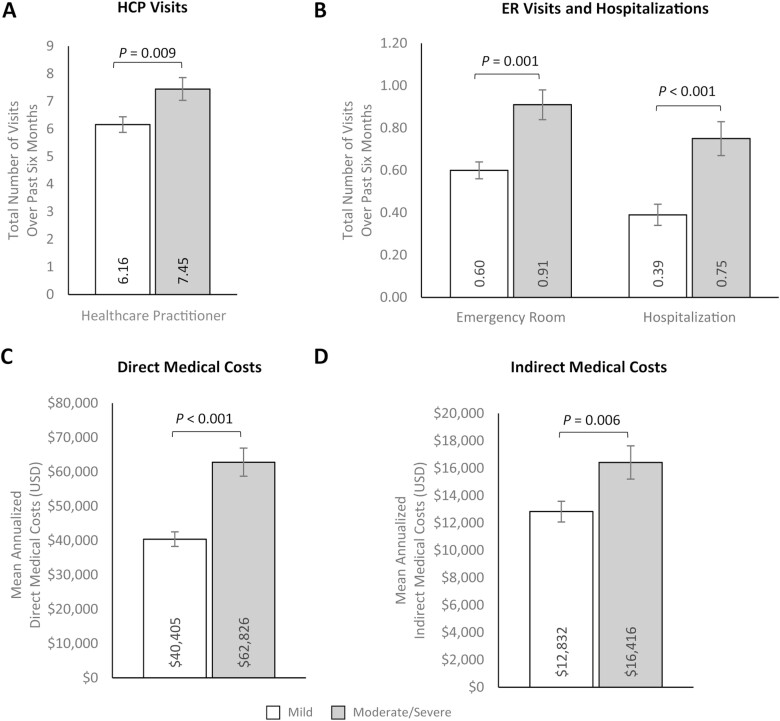
Multivariable analyses of (A) HCP visits, (B) ER visits and hospitalizations, (C) annualized direct medical costs, and (D) annualized indirect medical costs by Crohn’s disease severity. Error bars represent standard error. Indirect costs were only calculated for participants in the labor force at the time of the survey and who had a valid response (ie, non-missing) for the number of hours worked over the past 7 days and the number of hours missed in the past 7 days (mild *n* = 425; moderate/severe *n* = 274). Outcomes were modeled using a log link with a negative binomial distribution; exp(β) = rate ratio. Models control for age (continuous; set to mean = 45.19 years), gender (male [ref]; female), marital status (single/never married/decline to answer; married/living with a partner [ref]), educational attainment (less than a college degree/declined to answer; college graduate or higher [ref]), household income (<$25 000; $25 000 to <$50 000; $50 000 to <$100 000; $100 000 + [ref]), health insurance coverage (Medicare; Medicaid/VA/CHAMPUS; uninsured; commercial/other types of insurance [ref]), weight status (obese; overweight; underweight/normal weight/ declined to answer [ref]), smoking status (current smoker; former smoker; never smoker [ref]), alcohol use (drinks alcohol; does not drink alcohol [ref]), and Charlson Comorbidity Index Score (continuous; set to mean = 1.14). Abbreviations: ER, emergency room; HCP, healthcare practitioner; USD, United States dollars.

Additionally, participants with moderate/severe CD reported significantly higher HCRU than those with mild CD, with greater HCP visits (7.45 [SE: 0.41] vs. 6.16 [SE: 0.28]; *P* = .009), ER visits (0.91 [SE: 0.08] vs. 0.60 [SE: 0.05]; *P* = .001), and hospitalizations (0.75 [SE: 0.08] vs. 0.39 [SE: 0.04]; *P *< .001) over the past 6 months (Figure 3A and 3B, Supplementary Table S1). Consistent with these results, estimated mean direct medical costs were significantly higher among participants with moderate/severe CD ($62 826.29 [SE: $4110.50]) than participants with mild CD ($40 404.85 [SE: $2133.72]; *P* < .001; Figure 3C, Supplementary Table S1). Mean indirect costs (ie, costs related to workplace productivity impairment) were also significantly higher among participants with moderate/severe CD than participants with mild CD ($16 415.56 [SE: $1214.95] vs. $12 831.65 [SE: $755.57]; *P* = .006; Figure 3D, Supplementary Table S1). Multivariable results for outcomes by disease severity largely aligned with previous bivariate analyses (Supplementary Table S2).

### Study Outcomes by Race/Ethnicity—Main Effects

**Figure 4.**
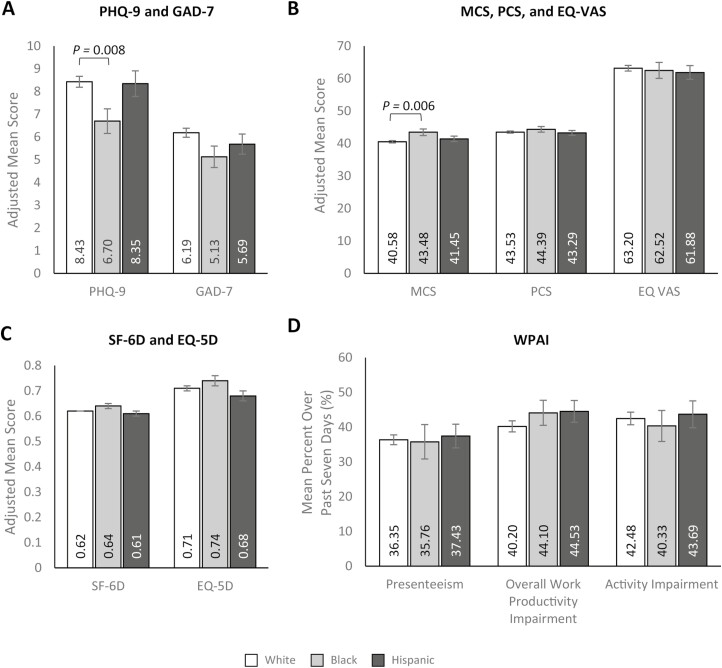
Multivariable analyses of patient-reported outcomes of (A) PHQ-9 and GAD-7, (B) MCS, PCS, and EQ VAS, (C) SF-6D and EQ 5D, and (D) WPAI by race/ethnicity. Lower PHQ-9 and GAD-7 scores and higher MCS, PCS, EQ VAS, SF-6D, and EQ-5D scores indicate better HRQoL. Error bars represent standard error. Presenteeism was only assessed among participants who worked > 0 hours over the last 7 days. PHQ-9, GAD-7, and WPAI outcomes were modeled using a log link with a negative binomial distribution; exp(β) = rate ratio. MCS, PCS, EQ VAS, SF-6D, and EQ-5D were modeled using an identity link with a normal distribution; β = estimated mean difference. Models control for age (continuous; set to mean = 45.19 years), gender (male [ref]; female), marital status (single/never married/decline to answer; married/living with a partner [ref]), educational attainment (less than a college degree/declined to answer; college graduate or higher [ref]), household income (<$25 000; $25 000 to <$50 000; $50 000 to <$100 000; $100 000 + (ref]), health insurance coverage (Medicare; Medicaid/VA/CHAMPUS; uninsured; commercial/other types of insurance [ref]), weight status (obese; overweight; underweight/normal weight/ declined to answer [ref]), smoking status (current smoker; former smoker; never smoker [ref]), alcohol use (drinks alcohol; does not drink alcohol [ref]), and Charlson Comorbidity Index Score (continuous; set to mean = 1.14). Abbreviations: EQ-5D-5L, EuroQol 5-dimension 5-level; GAD-7, generalized anxiety disorder—7 item; MCS, mental component summary; PCS, physical component summary; PHQ-9, patient health questionnaire-9 item; SF-6D, short form—6 dimension; WPAI, work productivity and activity impairment; VAS, visual analog scale.

In multivariable analyses adjusting for potential confounding variables, non-Hispanic Black participants with CD reported lower PHQ-9 (depression) scores than non-Hispanic White participants (6.70 [SE: 0.54] vs. 8.43 [SE: 0.24]; *P* = .008), indicating less depressive symptomology, whereas Hispanic participants had PHQ-9 scores that were comparable to non-Hispanic White participants (Figure 4A, Supplementary Table S3). There were no significant differences in GAD-7 (anxiety) scores between race/ethnicity groups (*P* > .05; Figure 4A, Supplementary Table S3). Estimated mean MCS scores were also significantly higher among non-Hispanic Black compared with non-Hispanic White participants, suggesting better mental health in the context of HRQoL (43.48 [SE: 0.99] vs. 40.58 [SE: 0.35]; MD: 2.90 [SE: 1.06]; *P* = .006; Figure 4B, Supplementary Table S3). There were no differences in other HRQoL measures across race/ethnicity groups (Figure 4B and 4C, Supplementary Table S3).

**Figure 5.**
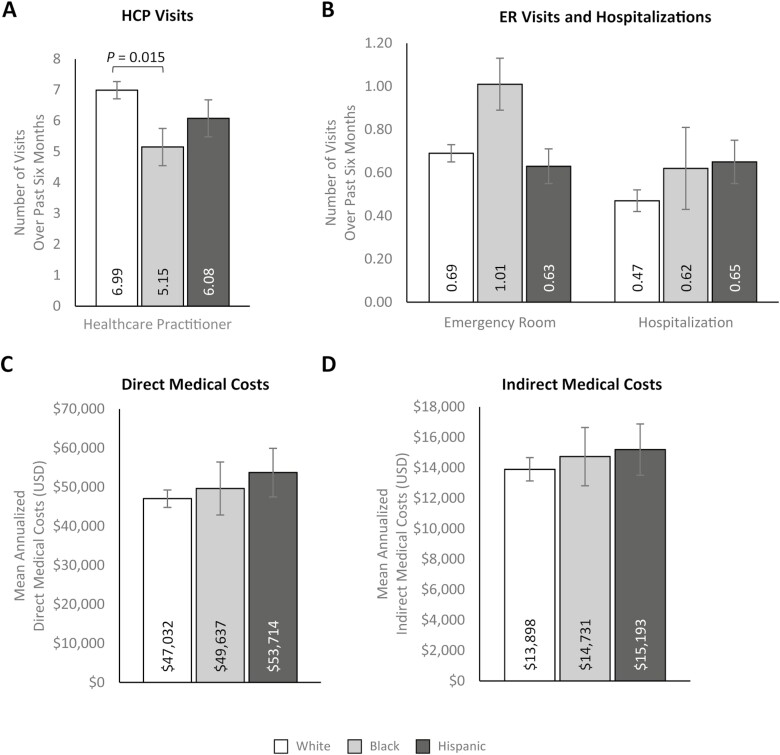
Multivariable analyses of (A) HCP visits, (B) ER visits and hospitalizations, (C) annualized direct medical costs, and (D) annualized indirect medical costs by Crohn’s disease severity. Note: Error bars represent standard error. Indirect costs were only calculated for participants in the labor force at the time of the survey and who had a valid response (ie, non-missing) for the number of hours worked over the past 7 days and the number of hours missed in the past 7 days (non-Hispanic White *n* = 501; non-Hispanic Black *n* = 79; Hispanic *n* = 119). Outcomes were modeled using a log link with a negative binomial distribution; exp(β) = rate ratio. Models control for age (continuous; set to mean = 45.19 years), gender (male [ref]; female), marital status (single/never married/decline to answer; married/living with a partner [ref]), educational attainment (less than a college degree/declined to answer; college graduate or higher [ref]), household income (<$25 000; $25 000 to <$50 000; $50 000 to <$100 000; $100 000 + [ref]), health insurance coverage (Medicare; Medicaid/VA/CHAMPUS; uninsured; commercial/other types of insurance [ref]), weight status (obese; overweight; underweight/normal weight/ declined to answer [ref]), smoking status (current smoker; former smoker; never smoker [ref]), alcohol use [drinks alcohol; does not drink alcohol [ref]), and Charlson Comorbidity Index Score (continuous; set to mean = 1.14).Abbreviations: ER, emergency room; HCP, healthcare practitioner; USD, United States dollars.

Labor force participation was comparable across race/ethnicity groups (Supplementary Table S3), and WPAI outcomes did not differ relative to race/ethnicity groups (Figure 4D, Supplementary Table S3). The estimated mean number of HCP visits reported over the past 6 months was significantly lower among non-Hispanic Black participants (5.15 [SE: 0.60]) than non-Hispanic White participants (6.99 [SE: 0.28]; *P* = .015; Figure 5A, Supplementary Table S3); there was no difference between Hispanic and non-Hispanic White participants. Moreover, there were no significant differences between race/ethnicity groups in the estimated mean number of ER visits or hospitalizations over the past 6 months (Figure 5B, Supplementary Table S3), though non-Hispanic Black participants reported a greater number of ER visits than non-Hispanic White and Hispanic participants. In line with these findings, direct and indirect medical costs were comparable across race/ethnicity groups (Figure 5C and 5D, Supplementary Table S3). Bivariate analyses by race/ethnicity are presented in Supplementary Table S4.

### Interaction Between Race/Ethnicity and Disease Severity

**Figure 6.**
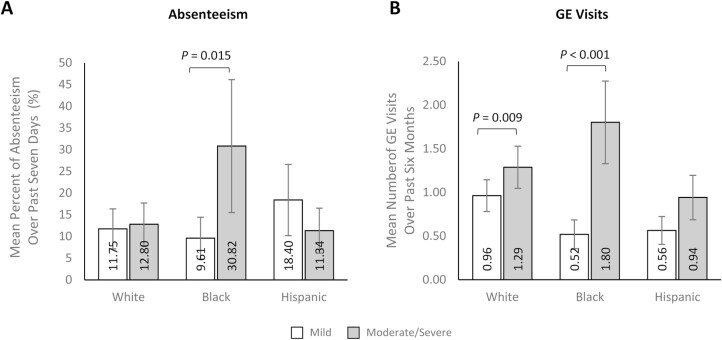
Multivariable analyses of absenteeism (A) and GE visits (B) stratified by race/ethnicity and condition severity. Note: Absenteeism was not calculated for those who worked 0 hours and missed 0 hours over the last 7 days. Error bars represent standard error. Results were modeled using the Likelihood Ratio Test. Models control for age (continuous; set to mean = 45.19 years), gender (male [ref]; female), marital status (single/never married/decline to answer; married/living with a partner [ref]), educational attainment (less than a college degree/declined to answer; college graduate or higher [ref]), household income (<$25 000; $25 000 to <$50 000; $50 000 to <$100 000; $100 000 + [ref]), health insurance coverage (Medicare; Medicaid/VA/CHAMPUS; uninsured; commercial/other types of insurance [ref]), weight status (obese; overweight; underweight/normal weight/ declined to answer [ref]), smoking status (current smoker; former smoker; never smoker [ref]), alcohol use (drinks alcohol; does not drink alcohol [ref]), and Charlson Comorbidity Index Score (continuous; set to mean = 1.14). Abbreviations: GE, gastroenterologist.

There was a significant interaction between race/ethnicity and CD severity on our multivariable models evaluating absenteeism and GE visits (Supplementary Table S5). In the main effects model, absenteeism was unrelated to both race/ethnicity and CD severity. However, race/ethnicity significantly moderated the relationship between CD severity and absenteeism (LRT X^2^_(2)_ = 7.11, *P *= .029), such that CD severity was significantly associated with absenteeism, but only among non-Hispanic Black participants. As such, non-Hispanic Black participants with moderate/severe CD reported significantly more absenteeism over the past 7 days than non-Hispanic Black participants with mild CD (30.82% [SE: 15.34] vs. 9.61% [SE: 4.80]; RR: 3.21 [SE: 1.54]; *P *= .015; Figure 6A).

Race/ethnicity also moderated the relationship between CD severity and GE visits over the past 6 months (LRT X^2^_(2)_ = 7.65, *P *= .022). The estimated mean number of GE visits was significantly higher among participants with moderate/severe CD than among those with mild CD for non-Hispanic White (1.29 [SE: 0.24] vs. 0.96 [SE: 0.18]; RR: 1.34 [SE: 0.15]; *P* = .009) and non-Hispanic Black (1.80 [SE: 0.47] vs. 0.52 [0.16]; RR: 3.46 [SE: 1.17]; *P* < .001) participants, and the magnitude of the difference was largest among non-Hispanic Black participants (Figure 6B).

## Discussion

This study aimed to assess PROs, including HRQoL and WPAI, and economic outcomes such as HCRU and annualized medical costs among 1,077 participants with CD relative to disease severity and race/ethnicity. Adjusted analyses showed that moderate/severe CD was associated with significantly worse outcomes than mild CD. We also found evidence to suggest that non-Hispanic Black race was associated with higher HRQoL scores with respect to depression and SF-36 MCS than non-Hispanic White race, and non-Hispanic Black race was associated with fewer HCP visits over the past 6 months than non-Hispanic White race. Finally, the relationship between CD severity and absenteeism, as well as the number of GE visits over the past 6 months, depended on race/ethnicity.

Participants with moderate/severe CD reported significantly worse HRQoL scores across all metrics measured. Importantly, the differences in SF-36 PCS (−4.84 [SE 0.56]) and EQ-5D-5L (−0.08 [SE 0.01]) between participants with moderate/severe CD and those with mild CD were clinically meaningful, highlighting the impact of CD symptom progression to a moderate/severe state. Participants with moderate/severe CD also reported greater work productivity impairment than those with mild CD, suggesting that more severe CD symptoms can significantly impact patients’ productivity and ability to remain in the workforce. Moderate/severe CD was also associated with higher HCRU, including HCP visits, ER visits, and hospitalizations; accordingly, participants with moderate/severe CD accumulated $22 421 more in annualized direct medical costs and $3584 more in annualized indirect costs than participants with mild CD. These results echo previous studies showing the association between greater CD severity and worse HRQoL, greater work impairment, increased HCRU, and higher medical costs.^7–10^ Furthermore, these results highlight the importance of widespread access to education, early management, and adequate treatment options to delay and/or prevent the progression of CD.

In our study, a greater proportion of non-Hispanic Black participants reported severe CD than non-Hispanic White participants. Additionally, non-Hispanic Black participants reported significantly fewer HCP visits over the past 6 months than non-Hispanic White participants. There were no statistically significant differences in any PROs between Hispanic and non-Hispanic White participants. Though not significant, non-Hispanic Black participants reported more ER visits and Hispanic participants reported more hospitalizations over the past 6 months than non-Hispanic White participants. Numerous studies establish that marginalized populations disproportionally encounter obstacles to receiving satisfactory care in healthcare systems.^27,28^ Delayed diagnosis among nonwhite patients is a primary concern, as IBD has traditionally been viewed as a disease of European populations.^14,29^ In fact, some studies have reported that non-Hispanic Black IBD patients have longer symptom durations and are older at the time of diagnosis than non-Hispanic Black individuals.^14,30^ Non-Hispanic Black CD patients also tend to have more severe disease phenotypes involving perianal and fistulizing disease, and are less likely to receive care from IBD specialists than similar non-Hispanic White patients.^29,31–33^ Furthermore, non-Hispanic Black and Hispanic patients with IBD experience more hospitalizations, longer hospital stays, and have a disproportionately higher rate of IBD-related mortality compared with non-Hispanic White patients.^34–36^ Altogether, these issues can contribute to more severe disease and worse health outcomes among non-Hispanic Black individuals. Consistently, in this study, the non-Hispanic Black cohort was the only group with a greater proportion of individuals with moderate/severe CD than mild CD. These issues may help explain our results of a greater proportion of non-Hispanic Black individuals with severe disease, as well as the trend toward more ER visits among the non-Hispanic Black cohort and more hospitalizations among the Hispanic cohort compared with non-Hispanic White participants.

Other potential contributors to greater disease severity, fewer HCP visits, and more ER visits observed among non-Hispanic Black participants may include decreased engagement with personal health, lack of access to healthcare resources, and distrust of the healthcare system.^37^ Indeed, even among our engaged study population, non-Hispanic Black participants had lower patient activation scores than other participants. Previous studies have shown that non-Hispanic Black populations in the United States have lower rates of commercial health insurance than non-Hispanic White populations, and non-Hispanic Black patients with IBD are more likely to report concerns about the cost of treatment than non-Hispanic White patients.^29,33^ Results from this study corroborate previous findings, with a greater proportion of non-Hispanic Black participants reporting a household income <$25 000 and lacking commercial health insurance compared with non-Hispanic White participants. Additionally, a higher proportion of non-Hispanic Black participants were single/unmarried than non-Hispanic White participants, which may influence financial stability and health insurance coverage. Out-of-pocket costs and the requirement to pay health insurance deductibles may also impede access to healthcare. Furthermore, recent evidence suggests that treatment with anti-TNF agents has a significantly lower therapeutic response among non-Hispanic Black patients with IBD than among non-Hispanic White patients, which may be due to the underrepresentation of racial/ethnic diversity in clinical trials.^38^ As anti-TNFs are the standard first-line treatment for many patients with moderate or severe IBD, the difference in therapeutic response may partially contribute not only to deferred healthcare access, but also to potential distrust of the healthcare system should concerns regarding lack of treatment effectiveness be dismissed. As such, as evidenced in this study, non-Hispanic Black participants may face financial barriers to healthcare access which prevent them from using primary and/or preventative care for their condition, instead turning to emergency care when their symptoms cannot be otherwise managed. Improved access and increasing racial/ethnic diversity of clinical trial participants may inform practice and offer potential solutions to some of the issues enumerated here.

Notably, in moderation analyses conducted to assess the relationship between CD severity and race on outcomes, the rate of workplace absenteeism and number of GE visits among non-Hispanic Black participants with moderate/severe CD was 3.21 and 3.46 times that of non-Hispanic Black participants with mild disease, respectively. In contrast, CD severity did not moderate any of the study outcomes among non-Hispanic White or Hispanic participants. Greater absenteeism among non-Hispanic Black participants with moderate/severe CD may be linked to experiencing more debilitating symptoms and the increased number of GE visits. Individuals who work in occupations that require an onsite presence or with inflexible schedules may find it impossible to work when their CD symptoms are severe. In a 2021 US Labor Force survey, employed non-Hispanic Black individuals were more likely than non-Hispanic White individuals to work in service occupations (21.5% vs. 15.0%) and production, transportation, and material moving (17.8% vs. 12.1%).^39^ Employed non-Hispanic Black individuals made up 12% of all employed workers but were substantially over-represented among detailed occupational categories of transit and intercity bus drivers (35%), nursing assistants (33%), security guards and gambling surveillance officers (33%) and home health aides (32%).^39^ Individuals in such roles may lack flexibility in their work schedule to attend healthcare provider visits and to continue working despite CD symptoms.^39^ Regarding GE visits, 1 possibility for the large magnitude of difference observed among the non-Hispanic Black participants is that, as previously discussed, social and financial barriers could prevent non-Hispanic Black individuals from visiting GE clinics until their symptoms become severe. In contrast, non-Hispanic White patients who do not experience these barriers may receive GE care even when their symptoms are milder. This may explain why we only observed a small difference in the number of GE visits between non-Hispanic White participants with mild or moderate/severe CD. Overall, these findings suggest severe CD symptoms may negatively influence work productivity and increase GE visits among non-Hispanic Black patients to a greater degree than other groups, and further underscores the importance of improved access and early diagnosis and treatment among non-Hispanic Black patients with CD.

Interestingly, non-Hispanic Black participants reported better HRQoL than non-Hispanic White participants with respect to depression (PHQ-9) and mental health (SF-36 MCS), whereas no differences in HRQoL were observed between Hispanic and non-Hispanic White participants. Overcoming the barriers experienced by non-Hispanic Black patients with CD during their healthcare journey, including delayed diagnoses, limited access, and different responses to treatments,^14,27–29,31–33^ as well as the marginalization and discrimination that non-Hispanic Black individuals experience within and outside of the healthcare sphere,^37,40^ may necessitate development/employment of a greater degree of adaptive social mechanisms to manage their health and well-being. Consistent with this idea, national studies have found that non-Hispanic Black individuals have a lower lifetime risk of psychiatric disorders, including depression and anxiety, than non-Hispanic White individuals, despite experiencing higher levels of social and financial adversity.^41^ As such, a number of studies have assessed factors that influence resilience to health conditions among non-Hispanic Black communities.^42^ However, it is important to recognize that in the context of equitable distribution of care, the need for patients to demonstrate or build resilience is a not desirable circumstance and may in fact indicate where improved equity and access to care is needed. It is also important to note that stigma surrounding mental illness among non-Hispanic Black communities may lead to apprehension in acknowledging mental health symptoms and seeking professional care; therefore, self-reported depressive and/or anxious symptomology among non-Hispanic Black participants could be underrepresented in this study.^43^

Because Hispanic individuals with IBD face many of the same barriers as non-Hispanic Black individuals, including delayed diagnosis, more hospitalizations, and higher rates of mortality, the absence of statistically significant differences between non-Hispanic White and Hispanic participants in this study is surprising.^14,34,35^ The pool of Hispanic participants surveyed may be heterogeneous with respect to ancestry and regional/cultural differences, and this may have limited the statistical power of analyses for the Hispanic group. Future analyses comparing non-Hispanic Black and Hispanic patients with CD may provide a better understanding of the differences between these 2 groups.

This study had several strengths including the use of self-reported disease severity and PROs, which provide patient perspectives on disease outcomes and are increasingly used in clinical studies over third-party perceptions of disease burden. The use of several different PROs to measure an outcome (eg, mental health) allowed us to capture a more holistic picture of health and well-being. Clinical trial study populations have not historically reflected the diversity and characteristics of the general/national disease population. As such, increasing racial/ethnic representation in PRO data collection can aid in understanding disease impacts on patient quality of life and help inform clinical care. The use of NHWS data, which is designed to be nationally representative of the US adult population with respect to age, sex, and race/ethnicity, in this study provides PRO data on a diverse group of patients with CD. Additionally, the sampling approach used by the NHWS ensures comprehensive capture of racial and ethnic groups, permitting comparisons between various races. The assessment of non-Hispanic Black and Hispanic groups allowed the identification of key differences between the 2 groups that would have been missed had these groups have been pooled together (ie, in a White vs. nonwhite comparison). The extensive roster of outcomes assessed in this study allows a robust contribution to the literature on a variety of PROs and economic outcomes, increases the validity of the study findings through consistency across measures, and contributes to a holistic understanding of patient burden in CD.

A key limitation of the study was that self-reported race and disease severity could not be independently confirmed since study information was collected via survey. However, the overall trend of participants with moderate/severe CD faring worse than participants with mild CD supports the validity of self-reported diagnosis data from the NHWS. Other limitations include the relatively small sample sizes for non-Hispanic Black and Hispanic groups, despite pooling data across 3 survey years, which may have limited the ability to detect significant associations across outcomes. With only 150 participants identifying as Hispanic, we were unable to compare any potential differences between Hispanic Whites and Hispanic Blacks due to insufficient power. As the survey was conducted in English in a virtual setting, certain populations could be underrepresented, including non-English speaking individuals, elderly groups, and those without reliable computer or internet access. Given that the NHWS is an internet-based convenience sample, our sample was generally more educated and affluent than adults in the general US population^44,45^; this difference was particularly pronounced among the non-Hispanic Black and Hispanic participants in our study. As such, the generalizability of our findings may be limited to a subset of patients with CD. While there are important generalizability limitations, our findings play a role in bridging the evidence gap, contributing to a better understanding of differences at various levels. Given the paucity of data, analyzing NHWS data is a step forward and further underscores the significant complexity and nuances. Another limitation is that cost data may underestimate the financial impact in the current post-COVID era, as medical costs were calculated using 2018 data. Residual confounding may have biased multivariable models, despite attempts to adjust for other potential explanatory variables. Finally, due to the cross-sectional nature of the study, causal inference was not possible.

## Conclusions

The study results demonstrate that patients with moderate/severe CD experience greater symptom burden than those with mild CD due to poor HRQoL, increased work productivity loss and activity impairment, greater HCRU, and higher medical costs. Race/ethnicity was associated with some outcomes, with non-Hispanic Black participants reporting higher HRQoL and lower HCP visits than non-Hispanic White participants. Further, non-Hispanic Black participants with severe CD reported greater absenteeism and more GE visits than non-Hispanic Black participants with mild CD. These findings emphasize the need for more effective treatments to reduce disease severity and highlight the importance of considering individual race/ethnicity groups rather than limiting comparisons to non-Hispanic White and grouped non-non-Hispanic White populations. Further research is required to better understand the potential factors influencing the relationship between CD severity and race/ethnicity among patients with CD. Studies like ours can aid in identifying racial/ethnic disparities in healthcare and support the development of resources and new approaches to reduce these inequities.

## Supplementary Material

otae021_suppl_Supplementary_Tables_S1-S5

## Data Availability

Data were obtained from NHWS through a data license agreement and are not publicly available.

## References

[1] Roda G , Chien Ng S, Kotze PG, et al. Crohn’s disease. Nat Rev Dis Primers. 2020;6(1):22. 32242028 10.1038/s41572-020-0156-2

[2] Kumar A , Cole A, Segal J, Smith P, Limdi JK. A review of the therapeutic management of Crohn’s disease. Therap Adv Gastroenterol. 2022;15:17562848221078456. 10.1177/17562848221078456 PMC8859667 35198041

[3] Dittrich AE , Sutton RT, Haynes K, Wang H, Fedorak RN, Kroeker KI. Incidence rates for surgery in Crohn’s disease have decreased: a population-based time-trend analysis. Inflamm Bowel Dis. 2020;26(12):1909–1916. 31895949 10.1093/ibd/izz315

[4] Bisgaard TH , Allin KH, Keefer L, Ananthakrishnan AN, Jess T. Depression and anxiety in inflammatory bowel disease: epidemiology, mechanisms and treatment. Nat Rev Gastroenterol Hepatol. 2022;19(11):717–726. 35732730 10.1038/s41575-022-00634-6

[5] van Gennep S , de Boer NKH, Gielen ME, et al. Impaired quality of working life in inflammatory bowel disease patients. Dig Dis Sci. 2021;66(9):2916–2924. 33063191 10.1007/s10620-020-06647-y PMC8379106

[6] Long GH , Tatro AR, Oh YS, Reddy SR, Ananthakrishnan AN. Analysis of safety, medical resource utilization, and treatment costs by drug class for management of inflammatory bowel disease in the united states based on insurance claims data. Adv Ther. 2019;36(11):3079–3095. 31562607 10.1007/s12325-019-01095-1 PMC6822802

[7] Moradkhani A , Beckman LJ, Tabibian JH. Health-related quality of life in inflammatory bowel disease: psychosocial, clinical, socioeconomic, and demographic predictors. J Crohns Colitis. 2013;7(6):467–473. 22884758 10.1016/j.crohns.2012.07.012

[8] Naegeli AN , Balkaran BL, Shan M, Hunter TM, Lee LK, Jairath V. The impact of symptom severity on the humanistic and economic burden of inflammatory bowel disease: a real-world data linkage study. Curr Med Res Opin. 2022;38(4):541–551. 35175166 10.1080/03007995.2022.2043655

[9] Reilly MC , Gerlier L, Brabant Y, Brown M. Validity, reliability, and responsiveness of the work productivity and activity impairment questionnaire in Crohn’s disease. Clin Ther. 2008;30(2):393–404. 18343277 10.1016/j.clinthera.2008.02.016

[10] Chudy-Onwugaje K , Mamunes AP, Schwartz DA, Horst S, Cross RK. Predictors of high health care utilization in patients with inflammatory bowel disease within 1 year of establishing specialist care. Inflamm Bowel Dis. 2021;27(3):325–335. 32488231 10.1093/ibd/izaa070 PMC7885330

[11] Molodecky NA , Soon IS, Rabi DM, et al. Increasing incidence and prevalence of the inflammatory bowel diseases with time, based on systematic review. Gastroenterology. 2012;142(1):46–54.e42; quiz e30.e42; quiz e30. 22001864 10.1053/j.gastro.2011.10.001

[12] Barnes EL , Nowell WB, Venkatachalam S, Dobes A, Kappelman MD. Racial and ethnic distribution of inflammatory bowel disease in the United States. Inflamm Bowel Dis. 2022;28(7):983–987. 34473272 10.1093/ibd/izab219

[13] Anyane-Yeboa A , Buadu MAE, Khalili H, Cozier YC. Epidemiology of inflammatory bowel disease in a cohort of US black women. Inflamm Bowel Dis. 2023;29(10):1517–1523. 36946376 10.1093/ibd/izad049 PMC11045662

[14] Liu JJ , Abraham BP, Adamson P, et al. The current state of care for black and hispanic inflammatory bowel disease patients. Inflamm Bowel Dis. 2023;29(2):297–307. 35816130 10.1093/ibd/izac124 PMC10210746

[15] Mercieca-Bebber R , King MT, Calvert MJ, Stockler MR, Friedlander M. The importance of patient-reported outcomes in clinical trials and strategies for future optimization. Patient Relat Outcome Meas. 2018;9:353-367. 30464666 10.2147/PROM.S156279 PMC6219423

[16] Farrell D , McCarthy G, Savage E. Self-reported symptom burden in individuals with inflammatory bowel disease. J Crohns Colitis. 2016;10(3):315–322. 26598526 10.1093/ecco-jcc/jjv218 PMC4957479

[17] Rubin DT , Siegel CA, Kane SV, et al. Impact of ulcerative colitis from patients’ and physicians’ perspectives: results from the UC: NORMAL survey. Inflamm Bowel Dis. 2009;15(4):581–588. 19067414 10.1002/ibd.20793

[18] Schreiber S , Panés J, Louis E, Holley D, Buch M, Paridaens K. Perception gaps between patients with ulcerative colitis and healthcare professionals: an online survey. BMC Gastroenterol. 2012;12:108. 22894661 10.1186/1471-230X-12-108 PMC3523079

[19] Enviza C. National Health and Wellness Survey (NHWS). https://www.cernerenviza.com/real-world-data/national-health-and-wellness-survey-nhws. Accessed: July 13, 2022.

[20] Hibbard JH , Stockard J, Mahoney ER, Tusler M. Development of the Patient Activation Measure (PAM): conceptualizing and measuring activation in patients and consumers. Health Serv Res. 2004;39(4 Pt 1):1005–1026. 15230939 10.1111/j.1475-6773.2004.00269.x PMC1361049

[21] Ware J , Kosinski M, Keller S. *SF-36 Physical and Mental Health Summary Scales: a User’s Manual* . 2001.

[22] Herdman M , Gudex C, Lloyd A, et al. Development and preliminary testing of the new five-level version of EQ-5D (EQ-5D-5L). Qual Life Res. 2011;20(10):1727–1736. 21479777 10.1007/s11136-011-9903-x PMC3220807

[23] Reilly MC , Zbrozek AS, Dukes EM. The validity and reproducibility of a work productivity and activity impairment instrument. PharmacoEcon. 1993;4(5):353–365. 10.2165/00019053-199304050-00006 10146874

[24] Agency for Healthcare Research and Quality. Number of people in thousands, United States, 1996-2018. Medical Expenditure Panel Survey.

[25] Dibonaventura M , Gupta S, McDonald M, Sadosky A. Evaluating the health and economic impact of osteoarthritis pain in the workforce: results from the National Health and Wellness Survey. BMC Musculoskelet Disord. 2011;12:83. 21527024 10.1186/1471-2474-12-83 PMC3110556

[26] Dibonaventura MD , Gupta S, McDonald M, Sadosky A, Pettitt D, Silverman S. Impact of self-rated osteoarthritis severity in an employed population: cross-sectional analysis of data from the national health and wellness survey. Health Qual Life Outcomes. 2012;10:30. 22420468 10.1186/1477-7525-10-30 PMC3342113

[27] Sewell JL , Velayos FS. Systematic review: the role of race and socioeconomic factors on IBD healthcare delivery and effectiveness. Inflamm Bowel Dis. 2013;19(3):627–643. 22623078 10.1002/ibd.22986 PMC3905682

[28] Sohn H. Racial and ethnic disparities in health insurance coverage: dynamics of gaining and losing coverage over the life-course. Popul Res Policy Rev. 2017;36(2):181–201. 28366968 10.1007/s11113-016-9416-y PMC5370590

[29] Anyane-Yeboa A , Li B, Traboulsi C, et al. Black race and public insurance are predictive of inappropriate evaluation of iron deficiency anemia and diarrhea. Dig Dis Sci. 2021;66(7):2200–2206. 32638203 10.1007/s10620-020-06434-9

[30] White JM , O’Connor S, Winter HS, et al. Inflammatory bowel disease in African American children compared with other racial/ethnic groups in a multicenter registry. Clin Gastroenterol Hepatol. 2008;6(12):1361-1369. 18848910 10.1016/j.cgh.2008.07.032 PMC3273485

[31] Coward S , Clement F, Benchimol EI, et al. Past and future burden of inflammatory bowel diseases based on modeling of population-based data. Gastroenterology. 2019;156(5):1345–1353.e4. 30639677 10.1053/j.gastro.2019.01.002

[32] Ng SC , Shi HY, Hamidi N, et al. Worldwide incidence and prevalence of inflammatory bowel disease in the 21st century: a systematic review of population-based studies. Lancet. 2017;390(10114):2769–2778. 29050646 10.1016/S0140-6736(17)32448-0

[33] Nguyen GC , LaVeist TA, Harris ML, Wang MH, Datta LW, Brant SR. Racial disparities in utilization of specialist care and medications in inflammatory bowel disease. Am J Gastroenterol. 2010;105(10):2202–2208. 20485281 10.1038/ajg.2010.202 PMC3170037

[34] Galoosian A , Rezapour M, Liu B, Bhuket T, Wong RJ. Race/ethnicity-specific disparities in in-hospital mortality and hospital charges among inflammatory bowel disease-related hospitalizations in the United States. J Clin Gastroenterol. 2020;54(7):e63–e72. 31008866 10.1097/MCG.0000000000001204

[35] Nguyen GC , Chong CA, Chong RY. National estimates of the burden of inflammatory bowel disease among racial and ethnic groups in the United States. J Crohns Colitis. 2014;8(4):288–295. 24074875 10.1016/j.crohns.2013.09.001

[36] Walker C , Allamneni C, Orr J, et al. Socioeconomic status and race are both independently associated with increased hospitalization rate among Crohn’s disease patients. Sci Rep. 2018;8(1):4028. 29507339 10.1038/s41598-018-22429-z PMC5838155

[37] Kennedy BR , Mathis CC, Woods AK. African Americans and their distrust of the health care system: healthcare for diverse populations. J Cult Divers. 2007;14(2):56–60. 19175244

[38] Burstiner LS , Owings AH, Royer A, et al. Black inflammatory bowel disease patients have lower response to antitumor necrosis factor agents compared with white patients. Inflamm Bowel Dis. 2023;29(12):1847-1853. 36808256 10.1093/ibd/izad005

[39] US Bureau of Labor Statistics. Labor force characteristics by race and ethnicity, 2021. https://www.bls.gov/opub/reports/race-and-ethnicity/2021/home.htm

[40] Banaji MR , Fiske ST, Massey DS. Systemic racism: individuals and interactions, institutions and society. Cogn Res Princ Implic. 2021;6(1):82. 34931287 10.1186/s41235-021-00349-3 PMC8688641

[41] Breslau J , Aguilar-Gaxiola S, Kendler KS, Su M, Williams D, Kessler RC. Specifying race-ethnic differences in risk for psychiatric disorder in a USA national sample. Psychol Med. 2006;36(1):57-68. 16202191 10.1017/S0033291705006161 PMC1924605

[42] Love MF , Brooks AN, Cox SD, et al. The effects of racism and resilience on Black stroke- survivor quality of life: Study protocol and rationale for a mixed-methods approach. Front Neurol. 2022;13:885374. 36034272 10.3389/fneur.2022.885374 PMC9399920

[43] Ward EC , Wiltshire JC, Detry MA, Brown RL. African American men and women’s attitude toward mental illness, perceptions of stigma, and preferred coping behaviors. Nurs Res. 2013;62(3):185–194. 23328705 10.1097/NNR.0b013e31827bf533 PMC4279858

[44] Bureau USC. Income in the United States: 2022. 2023. Accessed January 4, 2024, https://www.census.gov/library/publications/2023/demo/p60-279.html

[45] Bureau USC. Educational Attainment in the United States: 2021. 2022. Accessed January 4, 2024, https://www.census.gov/data/tables/2021/demo/educational-attainment/cps-detailed-tables.html

