# Supplementary material for: Racial and Ethnic Disparities in Health-Related Outcomes in Crohn’s Disease: Results From the National Health and Wellness Survey
Source: Crohns Colitis 360. 2024 Apr 12;6(2):otae021. doi: 10.1093/crocol/otae021 (PMC11041050; doi:10.1093/crocol/otae021)
Supplement: otae021_suppl_Supplementary_Tables_S1-S5 [file otae021_suppl_supplementary_tables_s1-s5.docx]

# Supplemental Materials

Study Outcome Definitions and Interpretation

Outcomes of interest included PROs of depression and anxiety, HRQoL, labor force participation, WPAI, HCRU, and medical costs. Depression severity was assessed using the Patient Health Questionnaire-9 (PHQ-9), a nine-item validated screening tool for measuring depression symptomology.^46^ Summed response scores range from 0 to 27. Anxiety severity was assessed using the Generalized Anxiety Disorder Assessment (GAD-7), a seven-item instrument that is used to measure the severity of generalized anxiety disorder (GAD).^47^ Summed response scores range from 0 to 21 for GAD-7. For both scales, higher scores indicate more severe depression or anxiety.

HRQoL was assessed using two summary scores of the Medical Outcomes Study 36-Item Short Form Survey Instrument (SF-36v2): physical component summary (PCS) and mental component summary (MCS).^21^ PCS and MCS scores range from 0 to 100, with higher scores indicating better quality of life. The minimum threshold for clinically important difference is 3.0 points. The SF-6D and the EuroQol 5-dimension health questionnaire (EQ-5D) instrument were used to assess health state utilities.^22^ The SF-6D utility scores were generated by applying the SF-36 algorithm to the SF-36v2. The SF-6D index yields summary scores ranging from 0 to 1, where higher scores indicate better quality of life and minimal clinically important difference [MCID] is defined as 0.033 points and minimal clinically important difference [MCID] is defined as 0.033 points. The EQ-5D instrument consists of the EQ-5D-5L utility index and EQ visual analog scale (VAS). The EQ-5D-5L utility index assesses HRQoL across five dimensions using a five-level rating scale, from no problems to extreme problems, with total scores ranging from -0.224 to 1 and an MCID of 0.028. The EQ VAS assesses patients’ self-rated health on a scale from 0 to 100, with the endpoints being ‘worst imaginable health state’ and ‘best imaginable health state’. Higher EQ-5D-5L and EQ VAS scores represent better HRQoL.

Labor force participation was derived from NHWS data through coding employment status as currently in the labor force (i.e., full-time employed, part-time employed, self-employed, or not unemployed but looking for work) or not currently in the labor force (i.e., retired, disabled, homemaker, student, or not employed and not looking for work). Work productivity was assessed using the WPAI questionnaire, a 6-item validated instrument measuring absenteeism (percentage of work time missed because of one's health), presenteeism (percentage of impairment experienced while at work because of one's health), overall work productivity loss (overall impairment estimate), and activity impairment (percentage of impairment in daily activities because of one's health) over the past seven days.^23^ Only participants who reported a work status of full-time, part-time, or self-employed provided data for absenteeism, presenteeism, and overall work impairment.

HCRU included the number of self-reported visits to any healthcare provider (HCP), gastroenterologists (GE), and emergency rooms (ER), and hospitalizations over the past six months. HCP visits include visits to any of the following: general practitioner/family practitioner, internist, allergist, cardiologist, dentist, dermatologist, diabetologist, endocrinologist, gastroenterologist, geriatrician, gynecologist, hepatologist, infectious disease specialist/infectologist, neurologist, nephrologist, nurse practitioner/physician assistant, obstetrician, oncologist, ophthalmologist, orthopedist, otolaryngologist, plastic surgeon, podiatrist, psychiatrist, psychologist/therapist, pulmonologist, respiratory therapist, rheumatologist, urologist, and other medical specialist. HCRU questions used the phrasing “for your own medical condition” to ensure that trips to accompany a friend or relative for their medical issues were not included in the calculation. The question phrasing was intentionally vague so that HCRU for any medical condition was included. Direct medical costs were imputed using data from the region-specific Medical Expenditure Panel Survey (MEPS) and included costs of an average HCP visit, ER visit, and hospitalization.^48^ Direct costs were calculated based on the annual number of visits, as estimated by doubling the number of visits self-reported over the past six months, and the average cost for each visit. Indirect costs were those associated with work productivity impairment and were calculated using estimated wages/salaries for each participant with data from the US Bureau of Labor Statistics. Mean absenteeism and presenteeism estimates were each multiplied by hourly wage rates to calculate total lost wages. Annual estimates of indirect costs assumed 50 work weeks in a year. This cost analysis approach has been used in prior research in the NHWS.^25,26^

Supplemental Table 1: Multivariable analyses of outcomes by severity among individuals with Crohn’s Disease

| **Outcomes** | **Race/Ethnicity** | **Adjusted Mean** | **Standard Error** | **95% CI for Adjusted Mean** | | **β or exp(β)** | **Standard Error** | **95% CI for β** | | ***P* value** |
| --- | --- | --- | --- | --- | --- | --- | --- | --- | --- | --- |
|  |  |  |  | **Lower** | **Upper** |  |  | **Lower** | **Upper** |  |
| **PHQ-9^a^** | Mild | 7.33 | 0.23 | 6.89 | 7.80 | ref | - | - | - | **-** |
|  | Moderate/severe | 9.78 | 0.38 | 9.07 | 10.55 | 1.34 | 1.05 | 1.21 | 1.47 | **<0.001** |
| **GAD-7^a^** | Mild | 5.55 | 0.21 | 5.16 | 5.96 | ref | - | - | - | **-** |
|  | Moderate/severe | 6.76 | 0.30 | 6.20 | 7.38 | 1.22 | 1.06 | 1.09 | 1.37 | **0.001** |
| **MCS^b^** | Mild | 42.14 | 0.39 | 41.37 | 42.91 | ref | - | - | - | **-** |
|  | Moderate/severe | 39.26 | 0.49 | 38.31 | 40.21 | -2.87 | 0.63 | -4.11 | -1.64 | **<0.001** |
| **PCS^b^** | Mild | 45.51 | 0.35 | 44.82 | 46.20 | ref | - | - | - | **-** |
|  | Moderate/severe | 40.67 | 0.43 | 39.83 | 41.52 | -4.84 | 0.56 | -5.94 | -3.73 | **<0.001** |
| **SF-6D^b^** | Mild | 0.64 | 0.01 | 0.63 | 0.65 | ref | - | - | - | **-** |
|  | Moderate/severe | 0.59 | 0.01 | 0.57 | 0.60 | -0.06 | 0.01 | -0.07 | -0.04 | **<0.001** |
| **EQ-5D^b^** | Mild | 0.74 | 0.01 | 0.73 | 0.76 | ref | - | - | - | **-** |
|  | Moderate/severe | 0.67 | 0.01 | 0.65 | 0.68 | -0.08 | 0.01 | -0.10 | -0.05 | **<0.001** |
| **EQ VAS^b^** | Mild | 66.62 | 0.98 | 64.70 | 68.53 | ref | - | - | - | **-** |
|  | Moderate/severe | 57.41 | 1.21 | 55.05 | 59.77 | -9.20 | 1.57 | -12.28 | -6.13 | **<0.001** |
| **Labor force participation^c^** | Mild | 71.8% | 2.1% | 67.5% | 75.9% | ref | - | - | - | **-** |
|  | Moderate/severe | 65.6% | 2.8% | 59.9% | 70.9% | 0.75 | 1.18 | 0.54 | 1.03 | 0.073 |
| **Absenteeism^a^** | Mild | 15.52 | 1.57 | 12.74 | 18.91 | ref | - | - | - | **-** |
|  | Moderate/severe | 17.46 | 2.15 | 13.71 | 22.22 | 1.12 | 1.17 | 0.83 | 1.53 | 0.451 |
| **Presenteeism^a^** | Mild | 32.63 | 1.52 | 29.78 | 35.74 | ref | - | - | - | **-** |
|  | Moderate/severe | 43.06 | 2.43 | 38.56 | 48.09 | 1.32 | 1.07 | 1.15 | 1.51 | **<0.001** |
| **Overall work productivity impairment^a^** | Mild  Moderate/severe | 36.96 | 1.76 | 33.66 | 40.58 | ref | - | - | - | **-** |
|  |  | 48.44 | 2.80 | 43.26 | 54.24 | 1.31 | 1.07 | 1.14 | 1.51 | **<0.001** |
| **Activity impairment^a^** | Mild | 37.13 | 1.08 | 35.07 | 39.32 | ref | - | - | - | **-** |
|  | Moderate/severe | 51.89 | 1.86 | 48.36 | 55.67 | 1.40 | 1.05 | 1.28 | 1.53 | **<0.001** |
| **HCP visits^a^** | Mild | 6.16 | 0.28 | 5.63 | 6.73 | ref | - | - | - | **-** |
|  | Moderate/severe | 7.45 | 0.41 | 6.68 | 8.31 | 1.21 | 1.08 | 1.05 | 1.40 | **0.009** |
| **GE visits^a^** | Mild | 0.56 | 0.04 | 0.48 | 0.64 | ref | - | - | - | **-** |
|  | Moderate/severe | 0.83 | 0.07 | 0.71 | 0.98 | 1.49 | 1.12 | 1.20 | 1.86 | **<0.001** |
| **ER visits^a^** | Mild | 0.60 | 0.05 | 0.51 | 0.71 | ref | - | - | - | **-** |
|  | Moderate/severe | 0.91 | 0.08 | 0.76 | 1.08 | 1.51 | 1.13 | 1.20 | 1.90 | **0.001** |
| **Hospitalizations^a^** | Mild | 0.39 | 0.04 | 0.32 | 0.47 | ref | - | - | - | **-** |
|  | Moderate/severe | 0.75 | 0.08 | 0.61 | 0.93 | 1.95 | 1.16 | 1.47 | 2.59 | **<0.001** |
| **Annualized direct medical costs^a^** | Mild | $40,404.85 | $2,133.72 | $36,431.98 | $44,810.95 | ref | - | - | - | **-** |
|  | Moderate/severe | $62,826.29 | $4,110.50 | $55,265.02 | $71,422.08 | 1.55 | 1.09 | 1.32 | 1.84 | **<0.001** |
| **Annualized indirect costs^a^** | Mild | $12,831.65 | $755.57 | $11,433.02 | $14,401.38 | ref | - | - | - | **-** |
|  | Moderate/severe | $16,415.56 | $1,214.95 | $14,198.96 | $18,978.19 | 1.28 | 1.09 | 1.07 | 1.53 | **0.006** |

^a^ Modeled using a log link with a negative binomial distribution; exp(β) = rate ratio.

^b^ Modeled using an identity link with a normal distribution; β = the estimated mean difference

^c^ Modeled using a logit link with a binomial distribution; exp(β) = odds ratio

Note: Models control for age (continuous; set to mean = 45.19 years), gender [male (ref); female], marital status [single/never married/decline to answer; married/living with a partner (ref)], educational attainment [less than a college degree/declined to answer; college graduate or higher (ref)], household income [<$25,000; $25,000 to <$50,000; $50,000 to <$100,000; $100,00+ (ref)], health insurance coverage [Medicare; Medicaid/VA/CHAMPUS; uninsured; commercial/TRICARE/don't know (ref)], weight status [obese; overweight; underweight/normal weight/ declined to answer (ref)], smoking status [current smoker; former smoker; never smoker (ref)], alcohol use [drinks alcohol; does not drink alcohol (ref)], and Charlson Comorbidity Index Score (continuous; set to mean = 1.14). Comparisons that reached statistical significance and highlighted in bold text.

Abbreviations: CD = Crohn’s Disease; EQ-5D-5L = EuroQol 5-Dimension 5-Level; ER = emergency room; GAD-7 = Generalized Anxiety Disorder – 7 item; GE = gastroenterologist; HCP = healthcare provider; HCRU = healthcare resource utilization; MCS = Mental Component Summary; PCS = Physical Component Summary; PHQ-9 = Patient Health Questionnaire – 9 item; SD = standard deviation; SF-6D = Short Form – 6 Dimension; WPAI = Work Productivity and Activity Impairment; VAS = Visual Analog Scale.

**Supplemental Table 2: Bivariate analyses of outcomes by severity among individuals with Crohn’s Disease**

|  | **Mild**  **(n = 648)** | | | **Moderate/severe**  **(n = 429)** | | | ***P* value** |
| --- | --- | --- | --- | --- | --- | --- | --- |
|  | **Valid n** | **Mean (SD)** | **Median (min, max)** | **Valid n** | **Mean (SD)** | **Median (min, max)** |  |
| **HRQoL**  PHQ-9^a^  GAD-7^b^  MCS^c^  PCS^c^  SF-6D^c^  EQ-5D  EQ VAS | 648  648  648  648  648  648  648 | 7.85 (7.01)  6.09 (5.91)  42.85 (11.59)  45.85 (9.57)  0.649 (0.146)  0.750 (0.188)  67.13 (24.58) | 6.00 (0.00, 27.00)  5.00 (0.00, 21.00)  43.52 (11.19, 65.85)  47.01 (20.02, 66.23)  0.640 (0.30, 1.00)  0.800 (-0.11, 1.00)  75.00 (1.00, 100.00) | 429  429  429  429  429  429  429 | 11.33 (7.34)  8.30 (6.11)  38.18 (11.25)  40.16 (9.55)  0.571 (0.123)  0.651 (0.224)  56.63 (26.78) | 11.00 (0.00, 27.00)  8.00 (0.00, 21.00)  36.59 (7.31, 68.99)  39.76 (10.60, 66.28)  0.560 (0.32, 0.96)  0.708 (-0.11, 1.00)  60.00 (1.00, 100.00) | **<0.001**  **<0.001**  **<0.001**  **<0.001**  **<0.001**  **<0.001**  **<0.001** |
| **Labor force participation, n (%)** | 648 | 425 (65.6%) | - | 429 | 274 (63.9%) | - | 0.563 |
| **WPAI**  Absenteeism^d^  Presenteeism^d^  Overall work productivity impairment  Activity impairment | 396  398  385  648 | 18.49 (26.18)  37.21 (33.17)  41.12 (36.05)  38.64 (31.76) | 2.00 (0.00, 100.00)  30.00 (0.00, 100.00)  32.00 (0.00, 100.00)  30.00 (0.00, 100.00) | 263  265  259  429 | 27.27 (27.90)  53.66 (31.56)  60.78 (33.76)  55.85 (27.84) | 20.00 (0.00, 100.00)  60.00 (0.00, 100.00)  72.00 (0.00, 100.00)  60.00 (0.00, 100.00) | **<0.001**  **<0.001**  **<0.001**  **<0.001** |
| **HCRU**  HCP visits^e^  GE visits  ER visits  Hospitalizations | 648  648  648  648 | 6.44 (7.43)  0.61 (1.05)  0.91 (3.15)  0.68 (2.27) | 4.00 (0.00, 66.00)  0.00 (0.00, 6.00)  0.00 (0.00, 66.00)  0.00 (0.00, 44.00) | 429  429  429  429 | 8.17 (11.79)  0.90 (1.44)  1.48 (2.83)  1.37 (2.79) | 5.00 (0.00, 133.00)  0.00 (0.00, 10.00)  0.00 (0.00, 25.00)  0.00 (0.00, 23.00) | **0.003**  **<0.001**  **0.002**  **<0.001** |
| **Costs**  Annualized direct medical costs  Annual indirect costs^f^ | 648  412 | $46,873.28  ($92,141.77)  $14,499.69  ($16,962.87) | $18,080.00  ($0.00, $1,716,292.00)  $8,330.00  ($0.00, $102,375.00) | 429  268 | $78,377.91  ($120,069.75)  $19,956.51  ($19,416.01) | $36,160.00  ($0.00, $1,033,816.00)  $13,160.50  ($0.00, $107,576.00) | **<0.001**  **<0.001** |

^a^ PHQ-9 includes nine items (range of 0 to 27) where higher scores indicate mores severe depression.

^b^ GAD-7 includes seven items (range 0 to 21) and a higher score indicates more severe general anxiety disorder.
^c^ Differences in 3 points on the norm-based component summary scores and 0.041 points on health utilities represent clinically meaningful differences.
^d^ Absenteeism was not calculated for those who worked zero hours and missed zero hours in the last seven days and presenteeism was only asked among those who worked more than zero hours in the last seven days.

^e^ Includes visits to any of the following: general practitioner/family practitioner; internist; allergist; cardiologist; dentist; dermatologist; diabetologist; endocrinologist; gastroenterologist; geriatrician; gynecologist; hepatologist; infectious disease specialist/infectologist (diseases such as HIV or hepatitis); neurologist; nephrologist; nurse practitioner/physician assistant; obstetrician; oncologist; ophthalmologist; orthopedist; otolaryngologist (ears, nose, and throat specialist); plastic surgeon; podiatrist; psychiatrist; psychologist/therapist; pulmonologist (lung specialist); respiratory therapist; rheumatologist; urologist; other medical specialist.

^f^ Total annual indirect costs were only calculated among respondents who were participating in the labor force at the time of the survey and who had a valid response (i.e., non-missing) for the number of hours working in the past 7 days and the number of hours missed in the past 7 days.

Note: Comparisons that reached statistical significance and highlighted in bold text. Chi-square tests were used to determine significant differences for categorical variables. ANOVA and independent sample *t* tests were used for continuous variables. *P* values (α=0.05) were provided for the omnibus test and pairwise testing between race/ethnicity subgroups. Statistical significance was adjusted for multiplicity using the Bonferroni correction for multiple comparisons. Results from bivariate analyses were used to identify covariates for the multivariable models (variables that were associated with the key independent variables or were of theoretical importance).

Abbreviations: CD = Crohn’s Disease; EQ-5D-5L = EuroQol 5-Dimension 5-Level; ER = emergency room; GAD-7 = Generalized Anxiety Disorder – 7 item; GE = gastroenterologist; HCP = healthcare provider; HCRU = healthcare resource utilization; MCS = Mental Component Summary; PCS = Physical Component Summary; PHQ-9 = Patient Health Questionnaire – 9 item; SD = standard deviation; SF-6D = Short Form – 6 Dimension; WPAI = Work Productivity and Activity Impairment; VAS = Visual Analog Scale.

**Supplemental Table 3: Multivariable analyses of outcomes by race/ethnicity among individuals with Crohn’s Disease**

| **Outcomes** | **Race/Ethnicity** | **Adjusted Mean** | **Standard Error** | **95% CI for Adjusted Mean** | | **β or exp(β)** | **Standard Error** | **95% CI for β** | | ***P* value** |
| --- | --- | --- | --- | --- | --- | --- | --- | --- | --- | --- |
|  |  |  |  | **Lower** | **Upper** |  |  | **Lower** | **Upper** |  |
| **PHQ-9^a^** | White | 8.43 | 0.24 | 7.97 | 8.91 | ref | - | - | - | **-** |
|  | Black | 6.70 | 0.54 | 5.72 | 7.84 | 0.79 | 1.09 | 0.67 | 0.94 | **0.008** |
|  | Hispanic | 8.35 | 0.56 | 7.32 | 9.53 | 0.99 | 1.08 | 0.86 | 1.15 | 0.905 |
| **GAD-7^a^** | White | 6.19 | 0.20 | 5.81 | 6.60 | ref | - | - | - | **-** |
|  | Black | 5.13 | 0.47 | 4.28 | 6.14 | 0.83 | 1.10 | 0.68 | 1.01 | 0.057 |
|  | Hispanic | 5.69 | 0.44 | 4.88 | 6.62 | 0.92 | 1.09 | 0.78 | 1.09 | 0.319 |
| **MCS^b^** | White | 40.58 | 0.35 | 39.88 | 41.27 | ref | - | - | - | **-** |
|  | Black | 43.48 | 0.99 | 41.54 | 45.41 | 2.90 | 1.06 | 0.82 | 4.98 | **0.006** |
|  | Hispanic | 41.45 | 0.85 | 39.78 | 43.12 | 0.87 | 0.94 | -0.97 | 2.72 | 0.355 |
| **PCS^b^** | White | 43.53 | 0.32 | 42.91 | 44.15 | ref | - | - | - | **-** |
|  | Black | 44.39 | 0.88 | 42.67 | 46.11 | 0.86 | 0.95 | -1.00 | 2.71 | 0.364 |
|  | Hispanic | 43.29 | 0.76 | 41.80 | 44.78 | -0.24 | 0.84 | -1.88 | 1.41 | 0.777 |
|  | White | 0.62 | 0.00 | 0.61 | 0.63 | ref | - | - | - | **-** |
| **SF-6D^b^** | Black | 0.64 | 0.01 | 0.62 | 0.66 | 0.02 | 0.01 | 0.00 | 0.05 | 0.089 |
|  | Hispanic | 0.61 | 0.01 | 0.59 | 0.63 | 0.00 | 0.01 | -0.03 | 0.02 | 0.726 |
|  | White | 0.71 | 0.01 | 0.70 | 0.73 | ref | - | - | - | **-** |
| **EQ-5D^b^** | Black | 0.74 | 0.02 | 0.70 | 0.78 | 0.03 | 0.02 | -0.01 | 0.07 | 0.165 |
|  | Hispanic | 0.68 | 0.02 | 0.65 | 0.71 | -0.03 | 0.02 | -0.07 | 0.01 | 0.091 |
|  | White | 63.20 | 0.88 | 61.48 | 64.92 | ref | - | - | - | **-** |
| **EQ VAS^b^** | Black | 62.52 | 2.45 | 57.71 | 67.33 | -0.69 | 2.64 | -5.87 | 4.49 | 0.795 |
|  | Hispanic | 61.88 | 2.12 | 57.72 | 66.04 | -1.32 | 2.34 | -5.91 | 3.27 | 0.572 |
| **Labor force participation^c^** | White | 68.4% | 2.0% | 64.3% | 72.3% | ref | - | - | - | **-** |
|  | Black | 75.8% | 4.9% | 65.0% | 84.0% | 1.44 | 1.33 | 0.83 | 2.51 | 0.196 |
|  | Hispanic | 69.8% | 5.0% | 59.3% | 78.6% | 1.07 | 1.30 | 0.64 | 1.77 | 0.803 |
|  | White | 15.10 | 1.42 | 12.56 | 18.17 | ref | - | - | - | **-** |
| **Absenteeism^a^** | Black | 23.00 | 4.95 | 15.09 | 35.06 | 1.52 | 1.27 | 0.96 | 2.42 | 0.075 |
|  | Hispanic | 18.93 | 3.40 | 13.31 | 26.93 | 1.25 | 1.22 | 0.85 | 1.85 | 0.254 |
|  | White | 36.35 | 1.59 | 33.37 | 39.60 | ref | - | - | - | **-** |
| **Presenteeism^a^** | Black | 35.76 | 3.60 | 29.36 | 43.55 | 0.98 | 1.12 | 0.79 | 1.22 | 0.879 |
|  | Hispanic | 37.43 | 3.14 | 31.75 | 44.13 | 1.03 | 1.10 | 0.86 | 1.23 | 0.747 |
| **Overall work productivity impairment^a^** | White | 40.20 | 1.80 | 36.83 | 43.89 | ref | - | - | - | **-** |
|  | Black | 44.10 | 4.48 | 36.14 | 53.83 | 1.10 | 1.12 | 0.88 | 1.36 | 0.402 |
|  | Hispanic | 44.53 | 3.88 | 37.55 | 52.82 | 1.11 | 1.10 | 0.92 | 1.33 | 0.279 |
| **Activity impairment^a^** | White | 42.48 | 1.12 | 40.35 | 44.73 | ref | - | - | - | **-** |
|  | Black | 40.33 | 2.99 | 34.88 | 46.64 | 0.95 | 1.08 | 0.81 | 1.11 | 0.516 |
|  | Hispanic | 43.69 | 2.76 | 38.60 | 49.44 | 1.03 | 1.07 | 0.90 | 1.18 | 0.688 |
|  | White | 6.99 | 0.28 | 6.45 | 7.56 | ref | - | - | - | **-** |
| **HCP visits^a^** | Black | 5.15 | 0.60 | 4.09 | 6.47 | 0.74 | 1.13 | 0.58 | 0.94 | **0.015** |
|  | Hispanic | 6.08 | 0.60 | 5.01 | 7.37 | 0.87 | 1.12 | 0.70 | 1.08 | 0.201 |
|  | White | 0.69 | 0.04 | 0.61 | 0.79 | ref | - | - | - | **-** |
| **GE visits^a^** | Black | 0.68 | 0.12 | 0.49 | 0.96 | 0.98 | 1.20 | 0.69 | 1.41 | 0.928 |
|  | Hispanic | 0.45 | 0.08 | 0.32 | 0.64 | 0.65 | 1.21 | 0.45 | 0.94 | **0.024** |
|  | White | 0.69 | 0.05 | 0.60 | 0.79 | ref | - | - | - | **-** |
| **ER visits^a^** | Black | 1.01 | 0.19 | 0.70 | 1.44 | 1.45 | 1.22 | 0.98 | 2.15 | 0.060 |
|  | Hispanic | 0.63 | 0.10 | 0.46 | 0.85 | 0.91 | 1.18 | 0.66 | 1.26 | 0.567 |
|  | White | 0.47 | 0.04 | 0.39 | 0.56 | ref | - | - | - | **-** |
| **Hospitalizations^a^** | Black | 0.62 | 0.14 | 0.40 | 0.96 | 1.32 | 1.28 | 0.82 | 2.12 | 0.258 |
|  | Hispanic | 0.65 | 0.12 | 0.46 | 0.93 | 1.39 | 1.22 | 0.94 | 2.04 | 0.097 |
| **Annualized direct medical costs^a^** | White | $47,031.69 | $2,246.28 | $42,828.84 | $51,646.97 | ref | - | - | - | **-** |
|  | Black | $49,636.78 | $6,772.10 | $37,990.21 | $64,853.83 | 1.06 | 1.16 | 0.79 | 1.41 | 0.715 |
|  | Hispanic | $53,714.30 | $6,228.05 | $42,795.20 | $67,419.39 | 1.14 | 1.14 | 0.89 | 1.47 | 0.302 |
| **Annualized indirect costs^a^** | White | $13,897.79 | $769.57 | $12,468.43 | $15,491.02 | ref | - | - | - | **-** |
|  | Black | $14,730.84 | $1,916.07 | $11,415.90 | $19,008.38 | 1.06 | 1.15 | 0.81 | 1.40 | 0.678 |
|  | Hispanic | $15,192.72 | $1,691.12 | $12,214.82 | $18,896.61 | 1.09 | 1.13 | 0.86 | 1.38 | 0.460 |

^a^ Modeled using a log link with a negative binomial distribution; exp(β) = rate ratio.

^b^ Modeled using an identity link with a normal distribution; β = the estimated mean difference

^c^ Modeled using a logit link with a binomial distribution; exp(β) = odds ratio

Note: Models control for age (continuous; set to mean = 45.19 years), gender [male (ref); female], marital status [single/never married/decline to answer; married/living with a partner (ref)], educational attainment [less than a college degree/declined to answer; college graduate or higher (ref)], household income [<$25,000; $25,000 to <$50,000; $50,000 to <$100,000; $100,00+ (ref)], health insurance coverage [Medicare; Medicaid/VA/CHAMPUS; uninsured; commercial/TRICARE/don't know (ref)], weight status [obese; overweight; underweight/normal weight/ declined to answer (ref)], smoking status [current smoker; former smoker; never smoker (ref)], alcohol use [drinks alcohol; does not drink alcohol (ref)], and Charlson Comorbidity Index Score (continuous; set to mean = 1.14). Comparisons that reached statistical significance and highlighted in bold text.

Abbreviations: CD = Crohn’s Disease; EQ-5D-5L = EuroQol 5-Dimension 5-Level; ER = emergency room; GAD-7 = Generalized Anxiety Disorder – 7 item; GE = gastroenterologist; HCP = healthcare provider; HCRU = healthcare resource utilization; MCS = Mental Component Summary; PCS = Physical Component Summary; PHQ-9 = Patient Health Questionnaire – 9 item; SD = standard deviation; SF-6D = Short Form – 6 Dimension; WPAI = Work Productivity and Activity Impairment; VAS = Visual Analog Scale.

**Supplemental Table 4: Bivariate analyses of outcomes by race/ethnicity among individuals with Crohn’s Disease**

|  | **White**  **(n = 818)** | | **Black**  **(n = 109)** | | **Hispanic**  **(n = 150)** | | ***P* value** | | | |
| --- | --- | --- | --- | --- | --- | --- | --- | --- | --- | --- |
|  |  |  |  |  |  |  | **Omnibus** | **Bonferroni-adjusted pairwise comparisons** | | |
|  |  |  |  |  |  |  |  | **White vs. Black** | **White vs. Hispanic** | **Black vs. Hispanic** |
|  | **Valid n** | **Mean (SD)** | **Valid n** | **Mean (SD)** | **Valid n** | **Mean (SD)** |  |  |  |  |
| **HRQoL**  PHQ-9^a^  GAD-7^b^  MCS^c^  PCS^c^  SF-6D^c^  EQ-5D  EQ VAS | 818  818  818  818  818  818  818 | 8.75 (7.40)  6.55 (6.16)  41.76 (11.94)  43.80 (10.29)  0.630 (0.144)  0.724 (0.206)  63.56 (24.95) | 109  109  109  109  109  109  109 | 8.33 (5.71)  6.68 (4.99)  40.96 (10.79)  44.16 (9.47)  0.612 (0.132)  0.720 (0.168)  60.54 (29.98) | 150  150  150  150  150  150  150 | 12.56 (7.23)  9.49 (5.83)  36.90 (9.90)  41.96 (8.16)  0.556 (0.123)  0.628 (0.233)  61.38 (28.27) | **<0.001**  **<0.001**  **<0.001**  0.092  **<0.001**  **<0.001**  0.381 | >0.99  >0.99  >0.99  >0.99  0.660  >0.99  0.765 | **<0.001**  **<0.001**  **<0.001**  0.111  **<0.001**  **<0.001**  >0.99 | **<0.001**  **0.001**  **0.020**  0.237  **0.005**  **0.001**  >0.99 |
| **Labor force participation, n (%)** | 818 | 501 (61.2) | 109 | 79 (72.5) | 150 | 119 (79.3) | **<0.001** | 0.072 | **<0.001** | 0.601 |
| **WPAI**  Absenteeism^d^  Presenteeism^d^  Overall work productivity impairment  Activity impairment | 469  471  459  818 | 1916 (26.28)  41.46 (34.06)  45.47 (36.72)  43.47 (31.59) | 78  77  77  109 | 25.97 (27.50)  40.13 (31.42)  50.03 (35.71)  44.77 (28.98) | 112  115  108  150 | 31.09 (28.64)  55.74 (30.00)  63.43 (32.09)  57.07 (29.66) | **<0.001**  **<0.001**  **<0.001**  **<0.001** | 0.115  >0.99  0.909  >0.99 | **<0.001**  **<0.001**  **<0.001**  **<0.001** | 0.590  **0.004**  **0.038**  **0.005** |
| **HCRU**  HCP visits^e^  GE visits  ER visits  Hospitalizations | 818  818  818  818 | 7.24 (9.87)  0.76 (1.18)  0.96 (2.18)  0.75 (1.99) | 109  109  109  109 | 6.02 (6.93)  0.87 (1.65)  1.45 (3.27)  1.02 (2.00) | 150  150  150  150 | 7.33 (8.61)  0.43 (1.05)  1.92 (5.63)  1.98 (4.44) | 0.430  **0.004**  **<0.001**  **<0.001** | 0.615  >0.99  0.327  0.889 | >0.99  **0.007**  **0.001**  **<0.001** | 0.807  **0.013**  0.647  **0.006** |
| **Costs**  Annualized direct medical costs  Annual indirect costs^f^ | 818  489 | $53,681 ($90,905)  $15,961 ($17,847) | 109  79 | $56,803 ($80,328)  $14,963 ($16,977) | 150  112 | $92, 636 ($169,448)  $20,852 ($19,775) | **<0.001**  **0.025** | >0.99  >0.99 | **<0.001**  **0.030** | **0.020**  0.081 |

^a^ PHQ-9 includes nine items (range of 0 to 27) where higher scores indicate mores severe depression.

^b^ GAD-7 includes seven items (range 0 to 21) and a higher score indicates more severe general anxiety disorder.
^c^ Differences in 3 points on the norm-based component summary scores and 0.041 points on health utilities represent clinically meaningful differences.
^d^ Absenteeism was not calculated for those who worked zero hours and missed zero hours in the last seven days and presenteeism was only asked among those who worked more than zero hours in the last seven days.

^e^ Includes visits to any of the following: general practitioner/family practitioner; internist; allergist; cardiologist; dentist; dermatologist; diabetologist; endocrinologist; gastroenterologist; geriatrician; gynecologist; hepatologist; infectious disease specialist/infectologist (diseases such as HIV or hepatitis); neurologist; nephrologist; nurse practitioner/physician assistant; obstetrician; oncologist; ophthalmologist; orthopedist; otolaryngologist (ears, nose, and throat specialist); plastic surgeon; podiatrist; psychiatrist; psychologist/therapist; pulmonologist (lung specialist); respiratory therapist; rheumatologist; urologist; other medical specialist.

^f^ Total annual indirect costs were only calculated among respondents who were participating in the labor force at the time of the survey and who had a valid response (i.e., non-missing) for the number of hours working in the past 7 days and the number of hours missed in the past 7 days.

Note: Comparisons that reached statistical significance and highlighted in bold text. Chi-square tests were used to determine significant differences for categorical variables. ANOVA and independent sample *t* tests were used for continuous variables. *P* values (α=0.05) were provided for the omnibus test and pairwise testing between race/ethnicity subgroups. Statistical significance was adjusted for multiplicity using the Bonferroni correction for multiple comparisons. Results from bivariate analyses were used to identify covariates for the multivariable models (variables that were associated with the key independent variables or were of theoretical importance).

Abbreviations: CD = Crohn’s Disease; EQ-5D-5L = EuroQol 5-Dimension 5-Level; ER = emergency room; GAD-7 = Generalized Anxiety Disorder – 7 item; GE = gastroenterologist; HCP = healthcare provider; HCRU = healthcare resource utilization; MCS = Mental Component Summary; PCS = Physical Component Summary; PHQ-9 = Patient Health Questionnaire – 9 item; SD = standard deviation; SF-6D = Short Form – 6 Dimension; WPAI = Work Productivity and Activity Impairment; VAS = Visual Analog Scale.

**Supplemental Table 5: Type III sum of squares/likelihood ratio test for interaction term (CD severity and race/ethnicity) among individuals with Crohn’s Disease**

| **Outcome** | **Test Statistic** | **DF** | ***P* value** |
| --- | --- | --- | --- |
| PHQ-9 | 0.73 | 2 | 0.696 |
| GAD-7 | 0.18 | 2 | 0.912 |
| MCS | 0.01 | 2 | 0.993 |
| PCS | 2.84 | 2 | 0.059 |
| SF-6D | 1.00 | 2 | 0.367 |
| EQ-5D-5L | 0.64 | 2 | 0.530 |
| EQ VAS | 0.44 | 2 | 0.642 |
| Labor force participation | 0.17 | 2 | 0.918 |
| Absenteeism | 7.11 | 2 | **0.029** |
| Presenteeism | 1.77 | 2 | 0.412 |
| Overall work productivity impairment | 2.19 | 2 | 0.335 |
| Activity impairment | 0.97 | 2 | 0.616 |
| HCP visits | 1.66 | 2 | 0.437 |
| GE visits | 7.65 | 2 | **0.022** |
| ER visits | 2.28 | 2 | 0.320 |
| Hospitalizations | 1.66 | 2 | 0.437 |
| Total annualized direct medical costs | 0.97 | 2 | 0.615 |
| Total annualized indirect costs | 0.04 | 2 | 0.979 |

Note: The statistical significance of the interaction term in linear regression models (i.e., identity link with a normal distribution) was assessed using the F statistics from the type III sum of squares. In logistic (logit link with binomial distribution) and negative binomial models (log link with negative binomial distribution) the Likelihood Ratio Test was used to assess the statistical significance of the interaction. Both tests conceptually compare the fully adjusted model (including the interaction term) to the model without the interaction term. Comparisons that reached statistical significance and highlighted in bold text.
Abbreviations: EQ-5D-5L = EuroQol 5-Dimension 5-Level; ER = emergency room; GAD-7 = Generalized Anxiety Disorder – 7 item; GE = gastroenterologist; HCP = healthcare provider; HCRU = healthcare resource utilization; MCS = Mental Component Summary; PCS = Physical Component Summary; PHQ-9 = Patient Health Questionnaire – 9 item; SD = standard deviation; SF-6D = Short Form – 6 Dimension; VAS = Visual Analog Scale.
